# Glucose-mediated insulin secretion is improved in FHL2-deficient mice and elevated FHL2 expression in humans is associated with type 2 diabetes

**DOI:** 10.1007/s00125-022-05750-1

**Published:** 2022-07-08

**Authors:** Jayron J. Habibe, Maria P. Clemente-Olivo, Torsten P. M. Scheithauer, Elena Rampanelli, Hilde Herrema, Mariska Vos, Arnout Mieremet, Max Nieuwdorp, Daniel H. van Raalte, Etto C. Eringa, Carlie J. M. de Vries

**Affiliations:** 1 Department of Medical Biochemistry, Amsterdam UMC, location University of Amsterdam, Amsterdam, the Netherlands; 2 Amsterdam Cardiovascular Sciences, Diabetes and Metabolism, University of Amsterdam, Amsterdam, the Netherlands; 3 Amsterdam Gastroenterology, Endocrinology and Metabolism, University of Amsterdam, Amsterdam, the Netherlands; 4 Department of Physiology, Amsterdam UMC, location Vrije Universiteit Amsterdam, Amsterdam, the Netherlands; 5 Department of Experimental Vascular Medicine, Amsterdam UMC, location University of Amsterdam, Amsterdam, the Netherlands; 6 Department of Internal Medicine, Diabetes Center, Amsterdam UMC, location Vrije Universiteit Amsterdam, Amsterdam, the Netherlands; 7 Department of Physiology, Cardiovascular Institute Maastricht, Maastricht, the Netherlands

**Keywords:** FHL2, Four and a half LIM domains protein 2, Gene expression, Glucose tolerance test, Glucose-stimulated insulin secretion, GSIS, GTT, MIN6, Pancreatic islets

## Abstract

**Aims/hypothesis:**

The general population is ageing, involving an enhanced incidence of chronic diseases such as type 2 diabetes. With ageing, DNA methylation of *FHL2* increases, as well as expression of the four and a half LIM domains 2 (FHL2) protein in human pancreatic islets. We hypothesised that FHL2 is actively involved in glucose metabolism.

**Methods:**

Publicly available microarray datasets from human pancreatic islets were analysed for FHL2 expression. In FHL2-deficient mice, we studied glucose clearance and insulin secretion. Gene expression analysis and glucose-stimulated insulin secretion (GSIS) were determined in isolated murine FHL2-deficient islets to evaluate insulin-secretory capacity. Moreover, knockdown and overexpression of FHL2 were accomplished in MIN6 cells to delineate the underlying mechanism of FHL2 function.

**Results:**

Transcriptomics of human pancreatic islets revealed that individuals with elevated levels of HbA_1c_ displayed increased FHL2 expression, which correlated negatively with insulin secretion pathways. In line with this observation, FHL2-deficient mice cleared glucose more efficiently than wild-type littermates through increased plasma insulin levels. Insulin sensitivity was comparable between these genotypes. Interestingly, pancreatic islets isolated from FHL2-deficient mice secreted more insulin in GSIS assays than wild-type mouse islets even though insulin content and islet size was similar. To support this observation, we demonstrated increased expression of the transcription factor crucial in insulin secretion, MAF BZIP transcription factor A (MafA), higher expression of GLUT2 and reduced expression of the adverse factor c-Jun in FHL2-deficient islets. The underlying mechanism of FHL2 was further delineated in MIN6 cells. FHL2-knockdown led to enhanced activation of forkhead box protein O1 (FOXO1) and its downstream genes such as *Mafa* and *Pdx1* (encoding pancreatic and duodenal homeobox 1), as well as increased glucose uptake. On the other hand, FHL2 overexpression in MIN6 cells blocked GSIS, increased the formation of reactive oxygen species and increased c-Jun activity.

**Conclusions/interpretation:**

Our data demonstrate that FHL2 deficiency improves insulin secretion from beta cells and improves glucose tolerance in mice. Given that FHL2 expression in humans increases with age and that high expression levels of FHL2 are associated with beta cell dysfunction, we propose that enhanced FHL2 expression in elderly individuals contributes to glucose intolerance and the development of type 2 diabetes.

**Data availability:**

The human islet microarray datasets used are publicly available and can be found on https://www.ncbi.nlm.nih.gov/geo/.

**Graphical abstract:**

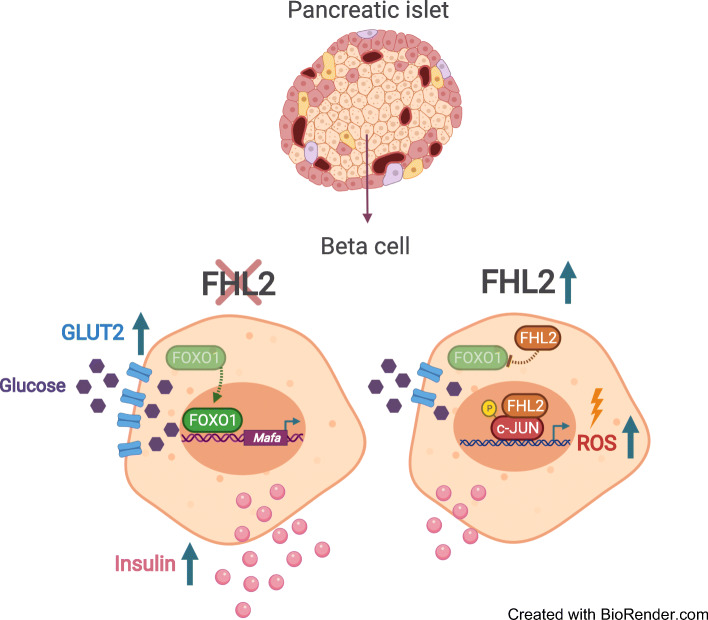

**Supplementary Information:**

The online version contains peer-reviewed but unedited supplementary material available at 10.1007/s00125-022-05750-1.

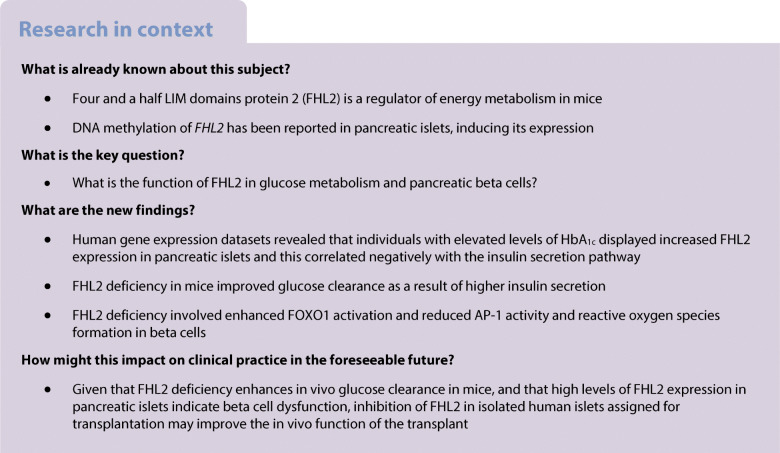



## Introduction

The global population is experiencing unprecedented ageing. The number of people aged over 60 years has doubled in the last 40 years [1] as a consequence of medical and social developments. However, the ageing process increases morbidities. These include deterioration of glucose metabolism, which at the onset involves insulin resistance and can lead to type 2 diabetes. The elderly population and individuals with obesity have the highest prevalence [2]. The exact underlying molecular mechanism causing deterioration of glucose metabolism is still elusive. The pathogenesis of type 2 diabetes starts when peripheral organs, such as adipose tissue and muscle, become resistant to insulin and are no longer able to take up circulating glucose. The hyperglycaemic state and reduction of insulin sensitivity impel the pancreatic beta cells from the islets of Langerhans to increase insulin secretion to maintain euglycaemia. When pancreatic beta cells fail to maintain the enhanced level of insulin secretion this results in type 2 diabetes. How this relative beta cell failure occurs is not yet completely understood. Recently, alterations in gene expression brought about by epigenetic changes have gained attention as one of the mechanisms underlying age-related pathologies. Interestingly, age-related DNA methylation changes in human blood cells and in pancreatic islets are associated with insulin secretion [3, 4]. Specific DNA methylation sites cause changes in the expression of genes such as *FHL2*, encoding for four and a half LIM domains 2 (FHL2). DNA methylation of *FHL2* in human pancreatic islets positively correlates with its expression [5]. Several forensic studies consistently found *FHL2* to be one of the DNA methylation markers that correlate most significantly with ageing [6–9].

FHL2 is composed of four complete LIM domains and each domain comprises two zinc finger structures and one additional zinc finger. FHL2 is expressed in different tissues and acts through protein–protein interactions. As a consequence, FHL2 regulates the activity and localisation of its interactors to fine-tune multiple intracellular signal transduction pathways. For instance, FHL2 is known to be a cofactor of several nuclear receptors, inhibiting or activating their transcriptional activity [10–12]. FHL2 was originally discovered in the heart and has been studied extensively in this organ. Additionally, FHL2 is expressed in different types of cancer; recently, we demonstrated that FHL2 deficiency protects mice from diet-induced obesity [13–15]. Despite low basal levels of FHL2 expression in the pancreas, previous finding by Bacos et al, showing that knockdown of FHL2 in INS-1e rat insulinoma cells decreases glucose-stimulated insulin secretion (GSIS), inspired us to study the role of FHL2 in glucose metabolism in more detail in human datasets of individuals with type 2 diabetes, in FHL2-deficient mice and in MIN6 cells.

In this study, we hypothesise a function for FHL2 in beta cells. Human microarray datasets from pancreatic islets were analysed for a potential correlation of FHL2 expression with insulin secretion. Next, we analysed the hyperglycaemic state after glucose administration in wild-type (WT) and FHL2-deficient mice and insulin secretion by isolated pancreatic islets. In addition, we modulated FHL2 expression in MIN6 cells to delineate its molecular mechanism in beta cells.

## Methods

Detailed descriptions of the experimental procedures can be found in the electronic supplementary material (ESM) Methods.

### Analysis of human pancreatic islet microarray datasets

Four public human islet microarray datasets (GSE38642, GSE54279, GSE76894 and GSE50397) were uploaded to the R2: Genomics Analysis and Visualization Platform (http://r2.amc.nl) [16–19]. Analysis of genes that associate with FHL2 expression was performed followed by pathway analysis. The gene lists were cross-referenced using http://bioinformatics.psb.ugent.be/webtools/Venn/. Based on the expression of the 85 genes in the insulin secretion Kyoto Encyclopedia of Genes and Genomes (KEGG) pathway, an average insulin secretion score was calculated.

### Animals

All animal experiments were approved by the ethics committee of the Amsterdam University Medical Center, the Netherlands, and performed under European directive 2010/63/EU guidelines. FHL2-deficient mice (*Fhl2*^−/−^) were generated by R. Bassel-Duby (University of Texas Southwestern Medical Center, Dallas, TX, USA) [14] and bred onto C57BL/6 background (Janvier-Labs, France, https://janvier-labs.com/en/fiche_produit/2_c57bl-6jrj_mouse/) for >11 generations. In all experiments, 8- to 22-week-old male littermates were used.

### Glucose and insulin tolerance

For the OGTT and IPGTT, mice were fasted before receiving an oral bolus or i.p. injection of glucose, respectively. For the ITT, fasted mice were given an i.p. injection of insulin. Blood samples were collected, centrifuged and plasma samples stored at −80°C.

### Immunofluorescence

Mouse pancreas was fixed, sectioned and stained with different primary antibodies and the corresponding Alexa-Fluor-conjugated antibodies.

### RNA extraction and quantitative real-time PCR

For quantitative real-time PCR (qPCR), total RNA was isolated using Trizol reagent (Invitrogen, USA) and gene expression was determined by qPCR using SensiFAST SYBR No-ROX Kit (Bioline, UK) on the LightCycler 480 II PCR platform (Roche, Switzerland). Primer sequences are shown in ESM Table 1.

### Cell culture and lentiviral transduction

Mouse insulinoma MIN6 cells’ origin and culture details can be found in detail in ESM Methods. Recombinant lentiviral particles encoding FHL2 with green fluorescent protein (GFP) tag and shRNA targeting mouse *Fhl2* (shFHL2) were produced, concentrated and titrated as described previously [7].

### Western blotting

Protein lysates were obtained using RIPA buffer and equal amounts were used to assess the level of expression of proteins.

### Thiazolyl blue tetrazolium bromide assay for cell proliferation

Cells were seeded and cultured in variable conditions to assess proliferation with the thiazolyl blue tetrazolium bromide (MTT) assay.

### Pancreatic islet isolation and GSIS

Mice were anaesthetised and underwent islet isolation as described previously [20]. GSIS experiments were performed as described previously [21]. Insulin secretion was measured using the mouse insulin ELISA kit (ALPCO 80-INSMS-E01; E10, USA) and normalised to total protein content.

### 2-(*N*-[7-nitrobenz-2-oxa-1,3-diazol-4-yl]amino)-2-deoxyglucose uptake assay and reactive oxygen species measurement in MIN6 cells

MIN6 cells (AddexBio no. C0018008, USA) were infected with recombinant lentiviral particles encoding FHL2 with GFP tag or shRNA targeting mouse *Fhl2*. To measure glucose uptake, cells were grown on coated glass coverslips and incubated with 2-(*N*-[7-nitrobenz-2-oxa-1,3-diazol-4-yl]amino)-2-deoxyglucose **(**2-NBDG) (Invitrogen, no. N13195, USA). To measure reactive oxygen species (ROS), cells were stained with CellROX Deep Red (Invitrogen, no. C10422, USA) and Hoechst 34580 (ThermoFisher, H21486) before fixation.

### Nuclear protein fractionation and AP-1 transcription factor subunit transcription activity assay

Nuclear proteins were extracted from MIN6 cells and the transcriptional activity of c-Jun was measured using TransAM (Active Motif, USA) according to the manufacturer’s protocol.

### Statistical analysis

Statistical analyses were performed using GraphPad Prism version 9.2.0 software. Data are presented as means ± SEM. *p* values were calculated using Student’s *t* test, one-way ANOVA or two-way ANOVA with Bonferroni post hoc correction if data were normally distributed. A *p* value <0.05 was considered statistically significant.

## Results

### FHL2 expression negatively correlates with insulin secretion in human pancreatic islets

**Fig. 1:**
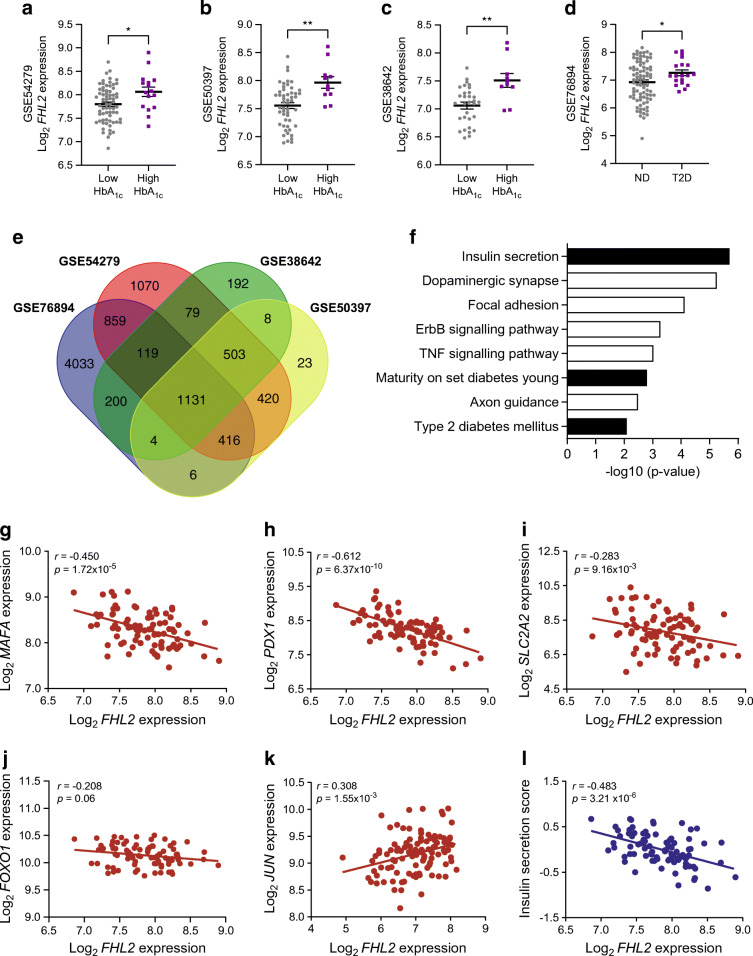
*FHL2* expression shows a negative correlation with the insulin secretion pathway in human pancreatic islets. (**a**–**d**) *FHL2* expression (Log_2_) in pancreatic islets of individuals with low (grey) or high (purple) HbA_1c_ levels in datasets GSE38642 (*n*=41; **a**), GSE54279 (*n*=84; **b**) and GSE50397 (*n*=62; **c**), and *FHL2* expression in islets of non-diabetic individuals (grey) and individuals with type 2 diabetes (purple) in dataset GSE76894 (*n*=103; **d**). (**e**) Venn diagram showing the number of genes that significantly correlate with FHL2 expression across all datasets. (**f**) Pathway analysis of the 1131 genes significantly correlated with FHL2 expression in all four datasets sorted by *p* value including epidermal growth factor (ErbB) and TNF signalling pathways. (**g**–**k**) Expression correlation of key insulin-secretory genes *MAFA* (**g**), *PDX1* (**h**), *SLC2A2* (**i**), *FOXO1* (**j**) from dataset GSE54279 and *JUN* from dataset GSE76894 (**k**) to *FHL2* expression. (**l**) Negative correlation of insulin secretion pathway signature score and *FHL2* expression in the GSE54279 dataset. Data are presented as mean ± SEM; *p<0.05 and **p<0.01. See ESM Fig. 1a–e for similar data from the other three datasets. ND, non-diabetic; T2D, type 2 diabetes

DNA methylation of *FHL2* increases with age and is associated with enhanced expression in human pancreatic islets and other tissues [5]. To determine whether *FHL2* expression is associated with specific genes and signalling pathways in human islets, we investigated human islet gene expression datasets. For three datasets [16–18], individuals were classified as either low HbA_1c_ or high HbA_1c_ according to blood HbA_1c_ levels. We observed higher *FHL2* expression levels in the high-HbA_1c_ group across all three datasets (Fig. 1a–c). In dataset GSE76894, the *FHL2* expression level was higher in individuals diagnosed with type 2 diabetes than in the non-diabetic individuals (Fig. 1d) [19]. Next, we performed enrichment analyses to identify genes for which the expression correlates positively or negatively with *FHL2* expression. Comparison of the results of all four datasets yielded a set of 1131 overlapping genes for which expression correlated with *FHL2* expression (Fig. 1e and ESM Table 2). Pathway analysis of these genes revealed ‘insulin secretion’ as the most represented, as well as other glucose-metabolism-related pathways including ‘maturity-onset diabetes of the young’ and ‘type 2 diabetes mellitus’ (Fig. 1f). To further explore the association of *FHL2* with insulin regulation and diabetes, we analysed the expression correlation of key insulin-secretory genes with *FHL2*. A negative correlation with *FHL2* expression was found for the solute carrier family 2 member 2 gene (*SLC2A2*, encoding GLUT2), pancreatic and duodenal homeobox 1gene (*PDX1*) and MAF BZIP transcription factor A (MafA) gene (*MAFA*), whereas expression of proinflammatory gene *JUN* increased concomitantly with *FHL2* expression and there was no consistent association with expression of the gene encoding for forkhead box protein O1 (FOXO1) (Fig. 1g–k). Finally, an overall insulin secretion pathway signature score was calculated for each of the low- and high-HbA_1c_ (or non-diabetic and type 2 diabetes) samples and the score was plotted against *FHL2* expression. These data further substantiated the negative association between *FHL2* expression and the insulin secretion pathway, indicating that high *FHL2* expression levels may counteract appropriate insulin release (Fig. 1l and ESM Fig. 1a–e).

### *Fhl2*^−/−^ mice show improved glucose clearance due to higher plasma insulin levels

**Fig. 2:**
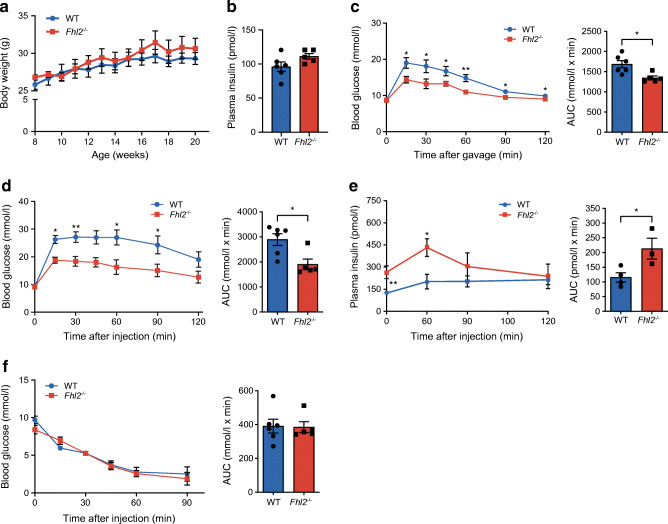
*Fhl2*^−/−^ mice have improved glucose tolerance and insulin secretion. (**a**) Body weight of WT and *Fhl2*^−/−^ mice during 14 weeks of standard chow diet. (**b**) Fasted plasma insulin of 8-week-old WT and *Fhl2*^−/−^ mice. (**c**) Blood glucose levels during OGTT in 16-week-old WT and *Fhl2*^−/−^ mice, with calculated AUCs. (**d**) Blood glucose levels during IPGTT in 20-week-old WT and *Fhl2*^−/−^ mice, with calculated AUCs. (**e**) Plasma insulin of mice during the IPGTT, with calculated AUCs. (**f**) Blood glucose levels during ITT in 22-week-old WT and *Fhl2*^−/−^ mice, with calculated AUCs. Data are presented as mean ± SEM; *p<0.05

To assess the functional involvement of FHL2 in glucose metabolism, we assayed the metabolic phenotype of male C57BL/6 WT mice and their *Fhl2*^−/−^ littermates. As previously reported, *Fhl2*^−/−^ mice do not show any specific features under normal conditions [13]. The body weight of mice aged 8–22 weeks fed a standard chow diet was similar when comparing WT and *Fhl2*^−/−^ mice (Fig. 2a); this is in line with similar food intake and organ weight after harvest at 22 weeks (ESM Fig. 2a,b). Fasting plasma insulin levels at the age of 8 weeks were also not different between groups (Fig. 2b). We performed an OGTT with 16-week-old mice. A marked increase in glucose clearance was observed in *Fhl2*^−/−^ mice compared with WT mice (Fig. 2c). The AUC of the OGTT showed a significant reduction in the blood glucose levels of *Fhl2*^−/−^ mice, indicating a more efficient glucose clearance from plasma. To discard any incretin effects on blood glucose levels, we repeated the GTT but injected the glucose intraperitoneally (IPGTT) and again measured blood glucose over time. The IPGTT also showed improved blood glucose clearance in *Fhl2*^−/−^ mice compared with WT littermates, as calculated from the AUC (Fig. 2d). In blood samples that were drawn during the IPGTT, we measured plasma insulin levels and calculated total insulin release (Fig. 2e). The *Fhl2*^−/−^ mice showed higher circulating insulin levels during the IPGTT, possibly explaining their ameliorated glucose clearance. To determine whether the increased glucose tolerance was caused by increased insulin sensitivity, the mice were subjected to an ITT at the age of 22 weeks, wherein they received an i.p. injection of insulin, followed by measurements of blood glucose over time (Fig. 2f). No difference in insulin sensitivity was found between WT and *Fhl2*^−/−^ mice, indicating that peripheral insulin sensitivity is not affected by FHL2 deficiency. Finally, we also made use of an indirect calorimetry system to analyse other variables that might affect energy metabolism in these mice. Interestingly, we found that only locomotor activity was lower for *Fhl2*^−/−^ mice (ESM Fig. 2c–e). Based on these data, we conclude that FHL2-deficient mice clear glucose faster after a glucose stimulus, involving enhanced insulin release.

### Pancreatic islets from *Fhl2*^−/−^ mice secrete more insulin than WT mice

**Fig. 3:**
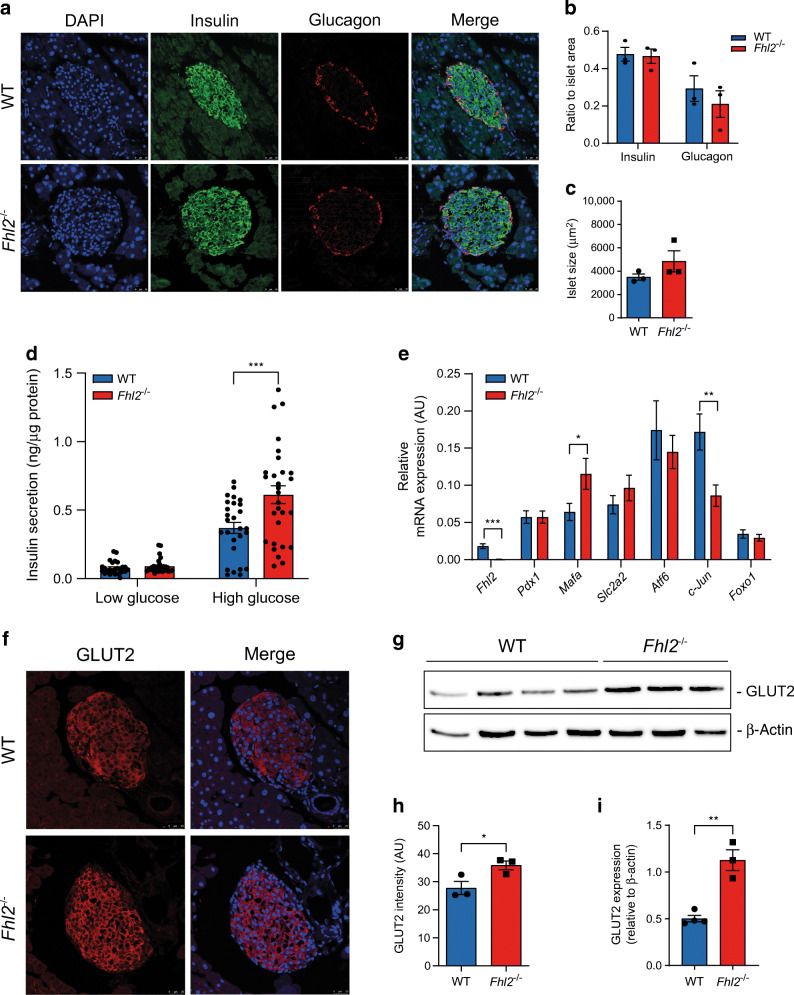
Pancreatic islets from *Fhl2*^−/−^ mice secrete more insulin and show changes in gene expression. (**a**) Nuclear DAPI (blue), insulin (green), glucagon (red) and merged immunofluorescent staining of pancreatic islets from WT and *Fhl2*^−/−^ mouse pancreas sections. (**b**) Quantification of insulin- and glucagon-positive area of islets (ratio of islet area) from WT and *Fhl2*^−/−^ mice (sections from *n*=3 mice per group). (**c**) Islet size (μm^2^) in WT and *Fhl2*^−/−^ mice (*n*>30 islets per group of three mice). (**d**) GSIS (at low [1.67 mmol/l] and high [16.7 mmol/l] glucose conditions) of islets isolated from WT (*n*=27) and *Fhl2*^−/−^ (*n*=29) mice. (**e**) Relative mRNA expression in arbitrary units (AU) of *Fhl2*, *Pdx1*, *Mafa*, *Slc2a2* (*Glut2*), *Atf6*, *c-Jun*, and *Foxo1* in pancreatic islets isolated from WT (*n*=17) and *Fhl2*^−/−^ (*n*=18) mice. (**f**) GLUT2 immunofluorescent staining (red), nuclear DAPI staining (blue) and merged staining of islets in pancreas sections from WT and *Fhl2*^−/−^ mice. (**g**) Representative western blot of GLUT2 in total pancreas lysates from WT (*n*=4) and *Fhl2*^−/−^ (*n*=3) mice. (**h**) Quantification of GLUT2 immunofluorescence intensity in arbitrary units (AU) of pancreatic islets (islets from *n*=3 mice per group). (**i**) Quantification of GLUT2 intensity relative to β-actin of western blot from (**g**). Data are indicated as mean ± SEM; *p<0.05, **p<0.01 and ***p<0.001

Given that *Fhl2*^−/−^ mice show an increased blood glucose clearance and higher plasma insulin levels compared with WT littermates, we decided to analyse islet size and insulin content in histological sections of the pancreas from both groups. Sections were stained for insulin and glucagon to localise the pancreatic islets and visualise the expression of these hormones within islets (Fig. 3a). Quantification of the staining, based on the immunofluorescence signal over the total islet area, revealed no difference in insulin or glucagon content when comparing islets from both groups (Fig. 3b). Average islet size and distribution were also similar (Fig. 3c and ESM Fig. 3a). To determine the insulin-secretory capacity of WT and *Fhl2*^−/−^ mouse islets, the islets were isolated and analysed with a GSIS assay. In line with our in vivo observations, there was no difference in insulin secretion under baseline conditions (low glucose), whereas upon high glucose stimulation, *Fhl2*^−/−^ mouse islets secreted significantly more insulin than WT mouse islets (Fig. 3d). We also determined the total insulin content of the islets (ESM Fig. 3b), which was similar for WT and *Fhl2*^−/−^ mice in agreement with the determination of insulin expression in the pancreatic sections (Fig. 3a,b). Next, the expression of genes crucial for islet function and GSIS was determined: *Pdx1*; *Mafa*; *Slc2a2* (*Glut2*); *ATF6*; *Jun*; and *Foxo1* (Fig. 3e). *Mafa* expression was increased in *Fhl2*^−/−^ mouse islets, whereas *Jun* expression was lower compared with WT mouse islets (Fig. 3e), in line with the human data presented in Fig. 1d. The expression of the other genes did not differ between the genotypes. Since *Slc2a2* (*Glut2*) is a reported MafA target gene [22], we decided to perform immunofluorescent staining to quantify GLUT2 protein levels. In islets of sections of the pancreas, we observed higher levels of GLUT2 protein expression in islets from *Fhl2*^−/−^ mice than in islets from WT mice (Fig. 3f,h). Next, GLUT2 protein was detected by western blotting in total pancreas lysates, confirming a 2.2-fold increased protein level of GLUT2 in the *Fhl2*^−/−^ mouse pancreas (Fig. 3g,i). To explore whether additional elements of the insulin-secretory pathway are affected by FHL2 deficiency, we determined mRNA expression of *Vamp2*, *Snap25*, *Stx1a*, *Trpm4*, *Kir6.2* (also known as *Kcnj11*) and *Gck* (ESM Fig. 3c). None of these genes showed significant regulation of expression by FHL2. Together, we conclude from these data that FHL2 deficiency in mouse islets induces a beneficial gene expression profile and enhanced GLUT2 expression with improved GSIS.

### FHL2 knockdown affects FOXO1 nuclear localisation and enhances the expression of FOXO1 target genes in MIN6 cells

**Fig. 4:**
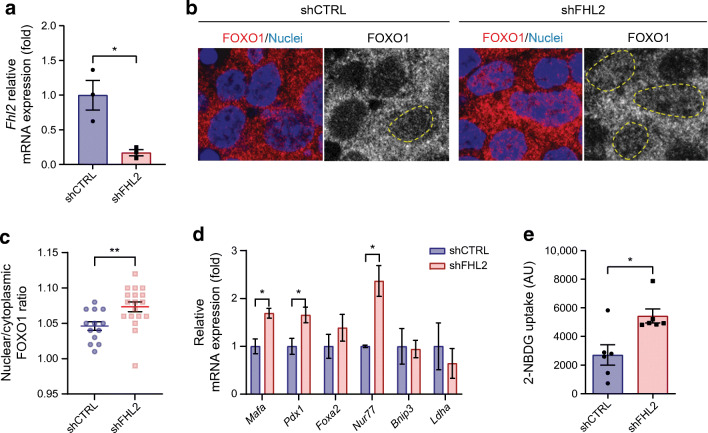
Knockdown of FHL2 in MIN6 cells leads to FOXO1 nuclear localisation and increased activity. (**a**) Relative mRNA expression of *Fhl2* in cells transduced with control shCTRL and shFHL2 (*n*=3). (**b**) FOXO1 (red) and nuclear DAPI (blue) immunofluorescent staining of shCTRL and shFHL2 cells; staining of FOXO1 alone is shown in grey, with FOXO1-positive nuclei indicated by yellow lines. (**c**) Quantification of FOXO1 staining as the ratio of nuclear over cytoplasmic intensity in MIN6 cells (*n*=13 and *n*=18 for shCTRL and shFHL2, respectively). (**d**) Relative mRNA expression of FOXO1 target genes (*Mafa*, *Pdx1*, *Foxa2*, *Nur77*, *Bnip3* and *Ldha*) in shCTRL and shFHL2 cells (*n*=3). (**e**) Uptake of fluorescently labelled glucose (2-NBDG) measured in arbitrary units (AU) by shCTRL and shFHL2 cells (*n*=6). Data are presented as mean ± SEM; *p<0.05 and **p<0.01

To unravel the underlying mechanism of improved GSIS by FHL2 deficiency, we initiated experiments in the insulinoma beta cell line MIN6. FOXO1 is a transcription factor involved in insulin secretion upstream of MafA and may protect cells against excessive ROS production [23]. FOXO1 activity is regulated through post-transcriptional modulation and its cellular localisation, shuttling between the cytoplasm and nucleus of beta cells. Interestingly, a link between FHL2 and FOXO1 has been described previously, indicating that FHL2 inhibits FOXO1 transcriptional activity through sirtuin 1 (SIRT1)-mediated deacetylation [24]. To determine whether FHL2 controls FOXO1 localisation and activity in beta cells, we performed FHL2 knockdown (shFHL2) experiments in MIN6 cells (Fig. 4a). FOXO1 was visualised by immunofluorescence and the subcellular localisation was analysed (Fig. 4b). Knockdown of FHL2 increased the FOXO1 nuclear/cytoplasmic ratio in MIN6 cells (Fig. 4c), indicating that FHL2 deficiency leads to increased nuclear localisation of FOXO1. To confirm that the nuclear localisation of FOXO1 indeed affects its transcriptional activity, we analysed the expression of known FOXO1 pancreatic target genes [25, 26]. Similar to the results in pancreatic islets, we confirmed that in shFHL2 cells, expression of *Mafa* was upregulated, as was *Pdx1* expression (Fig. 4d). Other FOXO1 target genes such as *Bnip3*, *Foxa2* and *Ldha* were not changed by FHL2 knockdown. *Nur77* (also known as *Nr4a1*) was strongly upregulated in shFHL2 cells; the encoded protein is reported to be a target of FOXO1 and is known to interact with FHL2. To assess whether regulation of GLUT2 protein expression by FHL2 deficiency, as shown in the pancreas (Fig. 3e), affects glucose uptake, MIN6 cells were exposed to fluorescently labelled deoxyglucose (with 2-NBDG). After FHL2-knockdown, glucose uptake was strongly increased (Fig. 4e). Altogether, FHL2 knockdown in MIN6 cells results in enhanced FOXO1 activity and increased glucose uptake, thus explaining the improved function of *Fhl2*^−/−^ mouse islets.

### FHL2 overexpression inhibits insulin secretion and enhances c-Jun activity and ROS accumulation in MIN6 cells

**Fig. 5:**
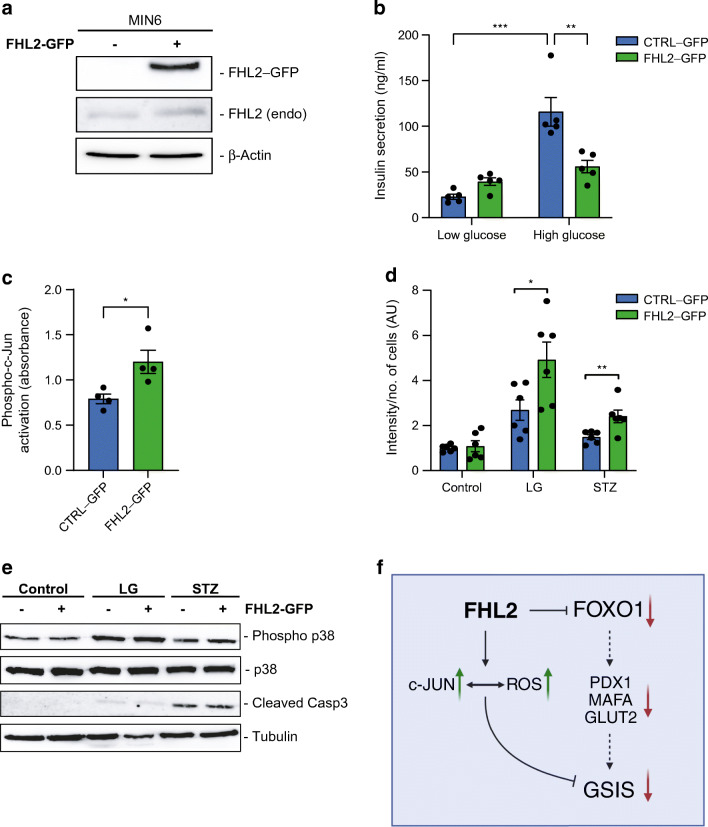
Overexpression of FHL2–GFP in MIN6 cells leads to inhibition of insulin secretion, activation of c-Jun and increased oxidative stress. (**a**) Representative western blot of FHL2 expression in control (CTRL–GFP) and FHL2–GFP-overexpressing MIN6 cells along with endogenous levels of FHL2 (endo). (**b**) GSIS of control and FHL2–GFP cells at low (1 mmol/l) and high (16 mmol/l) glucose conditions (*n*=5). (**c**) DNA binding activity of phosphorylated c-Jun (absorbance at 450 nm) in nuclear fractions from control and FHL2–GFP-overexpressing cells (*n*=4). (**d**) Intensity of ROS staining in arbitrary units (AU) in control and FHL2–GFP-overexpressing cells in regular medium, low glucose and 1 mmol/l streptozotocin (*n*=6). (**e**) Representative western blot of cells exposed to low glucose and streptozotocin for phosphorylated p38, total p38 and cleaved Casp3, and tubulin as a loading control. (**f**) Proposed mechanism underlying FHL2 function in beta cells. The graphical flowchart was created with BioRender.com. Data are indicated as mean ± SEM; *p<0.05, **p<0.01 and ***p<0.001. LG, low glucose; STZ, streptozotocin

Since we observed that FHL2 deficiency had a similar effect on isolated pancreatic islets and MIN6 cells, we wanted to explore the opposite situation and study the impact of stably enhanced expression of FHL2 in MIN6 cells. We applied lentiviral particles to overexpress FHL2 with a GFP tag (FHL2–GFP) in MIN6 cells (Fig. 5a). As we had already demonstrated that the lack of FHL2 was beneficial for beta cell function, here we hypothesised that FHL2 overexpression could be detrimental. In the GSIS assay we observed that overexpression of FHL2 indeed reduced the response of MIN6 cells to glucose almost completely (Fig. 5b). Given that FHL2 is known to interact with c-Jun [27], FHL2 expression is positively associated with c-Jun expression in humans (Fig. 1k) and c-Jun expression is decreased in FHL2-deficient isolated islets (Fig. 3e), we explored the impact of FHL2 on c-Jun activity in response to FHL2 overexpression in beta cells. c-Jun is one of the main subunits of the AP-1 transcription factor subunit (AP-1) complex, and we found increased AP-1 activity in FHL2–GFP cells based on the detection of phosphorylated c-Jun in the nuclear fraction bound to dsDNA comprising an AP-1-specific response element (Fig. 5c). These results are in line with a previous report showing that FHL2 is a coactivator of AP-1 through interaction with c-Jun and enhancement of its transcriptional activity [27]. AP-1 is known to activate several pathways, such as cell proliferation [28], so we performed an MTT assay to assess cell viability. The results were similar for FHL2-GFP and control cells (ESM Fig. 4), indicating that cell viability was not changed in response to FHL2 overexpression. Phosphorylation of c-Jun is mediated by activation of the mitogen-activated protein kinases (MAPK) pathway, which is triggered by multiple stimuli including ROS. Pancreatic beta cells are particularly sensitive to imbalances in redox homeostasis [29] and low glucose is sufficient to elicit an oxidative stress response [30], so we next studied the impact of FHL2–GFP overexpression in MIN6 cells on low glucose or streptozotocin exposure. After treating the cells, we measured intracellular ROS accumulation and discovered that FHL2–GFP cells contained higher amounts of ROS than control cells after such stimulation (Fig. 5d). We then analysed phosphorylation of MAPK p38, as well as the marker for apoptosis, cleaved caspase 3 (Casp3), to assess whether AP-1 activation triggered apoptosis (Fig. 5e). We observed an increase of phospho-p38 in low glucose culture and an increase in cleaved Casp3 with STZ stimulation but there was no difference between the FHL2–GFP and control cells. Taken together, these data show that overexpression of FHL2 strongly reduces GSIS in MIN6 cells, without affecting cell viability or apoptosis, at least partly due to enhanced ROS levels and AP-1 activity.

## Discussion

In this study, we determined that in transcriptome datasets of human pancreatic islets FHL2 expression is higher in individuals with increased HbA_1c_ values, as well as in those with type 2 diabetes. In addition, we revealed that FHL2 expression in human islets is negatively associated with genes instrumental for insulin secretion, such as *MAFA*, *PDX1* and *SLC2A2* (*GLUT2*) [31, 32]. Furthermore, *FHL2* mRNA levels are positively associated with the expression of *JUN*, a gene reported to suppress the insulin promoter and enhance ROS [33, 34]. Since glucose metabolism deteriorates with ageing, we hypothesise, based on these analyses, that in human pancreatic islets increased *FHL2* mRNA expression has subsequent negative consequences for islet function resulting in hampered insulin secretion. Our results show, however, a discrepancy with those published by Bacos et al [5], in which enhanced methylation of the *FHL2* gene was shown to associate with a lower risk for future type 2 diabetes. Our data seem controversial but different measures were compared: *FHL2* mRNA levels in pancreatic islets from individuals without and with high HbA_1c_ or diabetes [16–19] vs methylation status of the *FHL2* gene in blood in a follow-up study [5, 35].

We recently reported a novel role for FHL2 in the context of energy metabolism, as FHL2 deficiency protects mice from developing diet-induced obesity. Under those conditions, there were no differences in insulin sensitivity or glucose clearance between WT and *Fhl2*^−/−^ mice [15]. We now uncover that FHL2-deficient mice under standard-diet conditions handle glucose more efficiently than WT littermates and that this involves increased plasma insulin release upon glucose administration. We performed an OGTT as well as an IPTT, thus negating the incretin effect of the gut on glucose clearance, and still observed improved glucose clearance by the *Fhl2*^−/−^ mice. Furthermore, we analysed the morphological aspects of the pancreas and did not detect any difference in islet size, the ratio of alpha and beta cells, or insulin content when comparing WT and *Fhl2*^−/−^ mouse islets, leading us to propose that the ameliorated insulin secretion is due primarily to a difference in the molecular machinery underpinning insulin secretion rather than islet size. Similar to the diet-induced obesity experiment, we also did not observe changes in insulin sensitivity. It is worth mentioning that the mouse model applied is a full-body knockout and that we cannot attribute the phenotype we observe to a single organ or tissue. However, after isolating pancreatic islets from WT and *Fhl2*^−/−^ mice and stimulating the islets with high glucose we discovered that deleting *Fhl2* increases insulin release from the islets in concordance with corresponding changes in gene expression.

Intending to delineate the mechanism behind the phenotype observed in the mouse islets, we used the MIN6 insulinoma cell line. After knockdown of FHL2, we demonstrated that the transcription factor FOXO1, known to be inhibited via FHL2 [24], shows incremented nuclear localisation, when compared with control cells, and enhanced expression of FOXO1 target genes such as *Mafa*. Even though one of the main functions of MafA in beta cells is to promote insulin gene expression, which we did not find, MafA is also essential for GSIS and increased expression of MafA enhances cell glucose responsiveness [36, 37]. It is also important to mention that in the literature there are studies that show contradicting results for FOXO1 function in beta cells, either proposing a suppressive effect of FOXO1 on proliferation [38] or a protective effect through the increase of beta cell mass and against oxidative stress [39]. Our ex vivo mouse islet GSIS data showing increased insulin secretion by FHL2 deficiency, and the results obtained in MIN6 cells revealing that overexpression of FHL2 is detrimental for insulin secretion, are at odds with the data of Bacos et al, who showed (in rat-derived INS-1e cells only) that knockdown of FHL2 reduces insulin-secretory capacity [5]. In our hands, overexpression or knockdown of FHL2 was not effective in INS-1e cells, thus hampering the exact replication of those experiments. A possible explanation for this discrepancy could be intrinsic differences between these two cell lines, as reported previously [40, 41].

Since individuals with high HbA_1c_ levels express greater amounts of FHL2 in their pancreatic islets, we also investigated the effect of FHL2 overexpression in beta cells. We prove that overexpression of FHL2 is detrimental to beta cell function, as demonstrated by strong inhibition of GSIS. Previously, FHL2 was described as a coactivator of AP-1 [27] and c-Jun expression was downregulated in *Fhl2*^−/−^ mouse islets, prompting us to assess whether FHL2 might affect AP-1 activity. In MIN6 cells, we discovered that cells overexpressing FHL2 show a higher transcriptional activity of the AP-1 complex. One of the stimuli that cause AP-1 activation is oxidative stress and beta cells are remarkably vulnerable to changes in the amount of intracellular ROS since they express low levels of antioxidant enzymes [42]. Cells overexpressing FHL2–GFP indeed accumulate more ROS than control cells in stress conditions, although cell viability and apoptotic proteins are similar, indicating that FHL2 alters the cellular redox environment of beta cells. The exact function of FHL2 in this process, and its potential consequences, will require deeper characterisation but we propose that increased FHL2 expression in human pancreatic islets is to some extent involved in worsening beta cell function.

In conclusion, here we show a novel role of FHL2 in regulating both insulin secretion and glucose metabolism in vivo using FHL2-deficient mice and extensive human datasets. We determined that FHL2 deficiency improves pancreatic islet function and provides noteworthy improvement of whole-body glucose metabolism. The beneficial effect of FHL2 deficiency in mice likely also translates to humans based on our analyses of human islet datasets. Given the observation that FHL2 deficiency enhances GSIS and in vivo glucose clearance in mice, it makes sense that targeting FHL2 in pancreatic islets may serve as an effective strategy to combat beta cell dysfunction. Finally, high FHL2 expression levels in isolated human islets assigned for transplantation may indicate poor physiological quality of the transplant and lowering FHL2 expression could be an approach to improve the health of such islets.

## Supplementary Information

ESM (PDF 12378 kb)

## Data Availability

The human islet microarray datasets used are publicly available and can be found on https://www.ncbi.nlm.nih.gov/geo/. The data generated in the current study are available from the corresponding author upon reasonable request.

## Abbreviations

AP-1: AP-1 transcription factor subunit
Casp3: Caspase 3
FHL2: Four and a half LIM domains 2
FOXO1: Forkhead box protein O1
GSIS: Glucose-stimulated insulin secretion
MafA: MAF BZIP transcription factor A
MAPK: Mitogen-activated protein kinases
MTT: Thiazolyl blue tetrazolium bromide
PDX1: Pancreatic and duodenal homeobox 1
qPCR: Quantitative real-time PCR
ROS: Reactive oxygen species
WT: Wild-type
