# Supplementary material for: Glucose-mediated insulin secretion is improved in FHL2-deficient mice and elevated FHL2 expression in humans is associated with type 2 diabetes
Source: Diabetologia. 2022 Jul 8;65(10):1721–33. doi: 10.1007/s00125-022-05750-1 (PMC9477948; doi:10.1007/s00125-022-05750-1)
Supplement: Supplementary file 1 — (PDF 12378 kb) [file 125_2022_5750_MOESM1_ESM.pdf]

ELECTRONIC SUPPLEMENTARY MATERIAL (ESM)

ESM METHODS

Analysis of human pancreatic islet microarray datasets

Four publically accessible human islet microarray datasets GSE38642, GSE54279, GSE76894, and GSE50397 were uploaded to the R2: Genomics Analysis and Visualization Platform (http://r2.amc.nl) for meta-analysis [1–4]. We assessed FHL2 expression across these four human pancreatic islet micro-array datasets and split the subjects into two groups based on HbA1c values. Individuals with an HbA1c value of 6.0% or below were classified as 'Low HbA1c' while individuals with a value of 6.5% or higher were classified as 'High HbA1c'. This was done for all datasets except GSE76894, which contained individuals diagnosed with type 2 diabetes (T2D) as well as non-diseased (ND) individuals. Enrichment analysis for genes that associate with FHL2 expression was performed for all four datasets, and corrected for multiple testing via False Discovery Rate (FDR) yielding four gene lists along with correlation coefficients (R-value) and P-values for all the genes. Enrichment of pathways that associate with FHL2 expression in all four datasets was determined using KEGG pathway analysis. The gene lists were cross-referenced using the web tool http://bioinformatics.psb.ugent.be/webtools/Venn/. Insulin secretion pathway score was calculated for each sample within the High and Low HbA1c categories and we excluded samples with HbA1C between 6.0-6.5%, or that were missing HbA1c data. Based on the expression of the 85 genes defined in the insulin secretion KEGG pathway, an average insulin secretion score was calculated using R2 for each sample and plotted against the 2Log FHL2 expression.

Animals

All animal experiments were approved by the ethics committee of the Amsterdam University Medical Center, The Netherlands (permit number DBC287) and were performed in accordance with European directive 2010/63/EU guidelines. FHL2-deficient mice were generated by R. Bassel-Duby (University of Texas Southwestern Medical Center, Dallas, TX, USA) and were bred onto a C57BL/6 background (Janvier-Labs, France, https://janvier-labs.com/en/fiche_produit/2_c57bl-6jrj_mouse/) for more than 11 generations. In all experiments, male littermates of 8- to 22-week-old were used and *n* refers to the number of single animals. Sample size for each experiment was determined by power calculation using the nQuery software (https://www.statsols.com/). Littermates from wild-type and FHL2-deficient genotype were randomly separated in ventilated cages with free access to water and food (standard chow, Teklad Global #2016 diet, USA) and were euthanized by intraperitoneal injection of a lethal dose of ketamine (166 mg/kg) plus xylazine (24 mg/kg). Both number and suffering of animals were minimized as much as possible. Health was monitored weekly by researchers and animal caretakers and humane endpoints were accounted for under standard procedure from the animal research facility. After termination, mouse tissues were rinsed with ice-cold PBS through trans-cardiac perfusion, harvested and stored at -80ºC for further analysis.

Glucose and insulin tolerance

For both oral glucose tolerance tests (OGTT) and intraperitoneal glucose tolerance tests (IPGTT), mice were fasted 4 hours before receiving an oral glucose bolus (2 g/kg body weight) or intraperitoneal injection of glucose (2 g/kg body weight), respectively. For the insulin tolerance test (ITT), mice were fasted for 4 hours prior to injection of an intraperitoneal insulin dose (1 IU/kg body weight, Sigma Aldrich, USA, #I9278). In all experiments, whole-tail vein blood was collected at baseline and every 15 or 30 minutes for a period of 120 minutes, blood glucose was measured using an automatic StatStrip® glucose meter (Nova Biomedical, USA). At indicated time points blood samples from the tail vein were collected in EDTA-coated capillary tubes, centrifuged and plasma samples were stored at -80°C for

further insulin measurement (STELLUX® Chemi Rodent Insulin ELISA Jumbo #80-INSMR-CH10. USA). The age of the mice used for OGTT, IPGTT and ITT was 16, 20 and 22 weeks, respectively.

Immunofluorescence

Mouse pancreas was fixed in 4% paraformaldehyde (Roth, Denmark), embedded in paraffin, sectioned, and mounted on StarFrost glass slides (Thermo Scientific, USA). Sections were subjected to de-paraffinization, rehydration, treatment with 1% H_2O_2 (Merck, Germany) and subjected to heat-induced epitope retrieval with citrate buffer pH 6 at 95°C for 20 min . Sections from the head, body, and tail of the pancreas were stained for insulin (Thermofisher, PA1-26938, USA, 1:400), glucagon (Abcam, ab92517, UK, 1:500), GLUT2 (Abcam, ab54460, UK, 1:500), and FoxO1 (cell signaling, USA, C29H4, 1:100) and DAPI. Secondary antibodies used were Alexa-568 goat anti-Rabbit (Mol.Probes, USA) and Alexa-488 goat anti-Mouse both at 1:500 in 2% BSA in PBS. All antibodies were validated following manufacturer's instructions. Sections were visualized using The Leica TCS SP8 X confocal microscope and analyzed with LAS X 3D software.

Pancreatic islet isolation and (GSIS)

Male mice were anesthetized and sacrificed via intraperitoneal injection of 25 mg/mL of pentobarbital, and underwent pancreatic islet isolation as described previously [5]. Briefly, the common bile duct was clamped at the Ampulla of Vater and the pancreas was perfused with 3 ml cold collagenase solution (1000 U/ml Collagenase XI from Sigma Aldrich, USA, #C7657) prepared in Hanks' Balanced Salt Solution (GIBCO, USA #14185-052) and removed. Pancreata were digested for 13 minutes at 37°C then the digestion was stopped with ice-cold HBSS supplemented with 1 mM $CaCl_2$. Modified HBSS was added to the digestion solution and the pancreata were centrifuged, the supernatant was removed and the digested pancreata were washed twice with HBSS. Digested pancreata were forced through a 70µM strainer (BD Falcon, USA: 352350) into a 100mm petri dish containing RPMI 1640 medium (GIBCO #11875, USA) with L-glutamine, 10% Fetal Calf Serum (FCS), and 1% Pen Strep (P/S). Islets were handpicked and allowed to recover overnight in complete RPMI medium at 37°C and 5% CO_2 prior to experiments. Subsequent GSIS experiments were performed the next day as described previously [6]. The insulin secretion values were measured using the mouse insulin ELISA kit (ALPCO 80-INSMS-E01, E10) and normalized to total protein content of the islets.

RNA extraction and quantitative real-time PCR

Total RNA was isolated from islets and cells using Trizol reagent (Invitrogen, USA) according to the manufacturer's protocol. cDNA synthesis was performed using the iScript cDNA synthesis kit (BioRad, USA). Quantitative PCR was carried out using SensiFAST SYBR No-ROX Kit (Bioline, UK) on the LightCycler 480 II PCR platform (Roche). Cycle quantification and primer set amplification efficiency were calculated using the LinRegPCR software package. Target gene expression was normalized by dividing the geometric mean of the gene expression of 18s and 36B4 from mouse. Primer sequences used are listed in supplemental ESM Table 1.

Cell culture and lentiviral transduction

The clonal mycoplasma-free mouse insulinoma MIN6 cells (purchased from AddexBio, USA, #C0018008) were cultured in DMEM (Gibco #41965062) supplemented with 15% (v/v) FBS (Gibco #10270106), 100 U/mL penicillin and 100 mg/mL streptomycin, 1 mM sodium pyruvate (Gibco # 11360070), 10 mM HEPES (Gibco # 15630080) and 0.05 mM freshly added β-mercaptoethanol (Merck #8057400005) at 37°C and 5% CO_2. Recombinant lentiviral particles encoding FHL2 with GFP tag and

shRNA targeting mouse FHL2 were produced, concentrated, and titrated as described previously [7]. Cells were seeded at 70-80% confluency and incubated with recombinant lentivirus for 24 hours. After 24 hours, the medium was refreshed and cells were cultured using medium containing 1 µg/mL of the selection marker puromycin.

2-NBDG uptake assay and confocal imaging

MIN6 cells were grown on Poly-L-Lysine coated glass coverslips for 1 day in 24 wells plates at a density of $2x10^5$ cells/well. Media was removed and the cells were incubated with 17 µM of the fluorescent glucose analogue 2-NBDG (Invitrogen #N13195) diluted in KRB buffer at 37 °C for 15 minutes. The cells were then fixed with 4% paraformaldehyde, stained for DAPI and the glass coverslips were mounted on StarFrost glass slides with Mowiol. MIN6 cells were visualized using the Leica TCS SP8 X confocal microscope and analyzed with LAS X 3D software.

Western blotting

Protein was isolated using RIPA buffer (150 mM NaCl, 50 mM Tris pH 7.4, 1% Nonidet-P40, 0.5% sodium deoxycholate, 0.1% SDS, Roche Complete™ protease inhibitor cocktail, and PhosSTOP™ phosphatase inhibitor tablets) and quantified using the DC protein assay (BioRad). Equal amounts of protein lysate were loaded onto 12% SDS-PAGE gels along with the protein ladder standard (Precision Plus Protein™ All Blue Pre-stained Protein Standards from Bio Rad) and transferred to nitrocellulose membranes. Membranes were blocked with 5-10% non-fat milk for 1 hour and subsequently incubated with primary antibody at 4°C overnight (mouse anti-FHL2; Invitrogen #MA1-40200, rabbit anti-Cleaved Caspase-3; Cell Signaling #9661, rabbit anti-Phospho-p38 MAPK (Thr180/Tyr182); Cell Signaling #9211, rabbit anti-p38 MAPK; Cell Signaling #9212, mouse anti-Alpha-Tubulin; Cedarlane, Canada, #CLT9002 and rabbit anti-Beta-Actin; Cell Signaling #4967). Membranes were washed and incubated with appropriate HRP-conjugated secondary antibodies for 1 hour at room temperature and protein bands were visualized using Supersignal West Pico PLUS Chemiluminescent Substrate (Thermo Scientific) and ImageQuant LAS 4000 imager (GE Healthcare, USA).

Nuclear protein fractionation and transcription activity assay

Nuclear proteins were extracted from MIN6 cells overexpressing control and FHL2-GFP using the Nuclear extraction kit (Active Motif). Protein concentration was determined using the DC protein assay (BioRad). Transcriptional activity assay of c-Jun was measured using TransAM™ (Active Motif, USA) according to the manufacturer's recommended protocol. Briefly, nuclear extract (10 µg total protein) was added to oligonucleotide (5'-TGAGTCA—3')-coated wells. After incubation and washing, phospho-Ser73-c-Jun was bound with a specific antibody and subsequently detected with HRP-conjugated antibody and developing solution. Absorbance was determined at 450 nm with a reference wavelength of 650 nm.

Measurement of reactive oxygen species (ROS)

Cells were plated in high-binding 96 well plates (Greiner Bio-One #655097, Austria) after coating with poly-L-lysine. Cells were then incubated with low glucose medium and STZ (1 mM) as described before for 6 and 24 hours. Afterwards, cells were stained with 5 µM CellROX Deep Red (Invitrogen #C10422) and nuclear-counter staining Hoechst 34580 (ThermoFisher, H21486) for 30 minutes prior to fixation. Cells were imaged using the ImageXpress® Pico Automated Cell Imaging System and the intensity of staining was analyzed using Image Acquisition and Analysis Software CellReporterXpress (Molecular Devices, USA).

MTT assay for cell proliferation

Cells were seeded in 96-well plates and cultured in variable conditions. Then, cells were treated with thiazolyl blue tetrazolium bromide (MTT) at 0.5 mg/ml and incubated for 3 hours at 37°C. MTT-containing medium was removed and isopropanol was added to solubilize the formazan product. Absorbance was measured at 590 nm with a reference wavelength of 650 nm.

Statistical analysis

Statistical analyses were performed using GraphPad Prism version 9.2.0 software (https://www.graphpad.com/scientific-software/prism/). Data are presented as means ±SEM. P-values were calculated using Student's t -test, one-way ANOVA, or two-way ANOVA with Bonferroni post-hoc correction if data were normally distributed. In cases where data were not normally distributed we employed Mann-Whitney U test or Kruskal Wallis, and if variances were unequal Welch's correction was performed instead. A p-value < 0.05 was considered statistically significant.

1. Fadista J, Vikman P, Laakso EO, et al (2014) Global genomic and transcriptomic analysis of human pancreatic islets reveals novel genes influencing glucose metabolism. Proc Natl Acad Sci U S A , *111*(38), 13924-13929. https://doi.org/10.1073/pnas.1402665111
2. Krus U, King BC, Nagaraj V, et al (2014) The complement inhibitor CD59 regulates insulin secretion by modulating exocytotic events. Cell Metab, *19*(5), 883-890. https://doi.org/10.1016/j.cmet.2014.03.001
3. Solimena M, Schulte AM, Marselli L, et al (2018) Systems biology of the IMIDIA biobank from organ donors and pancreatectomised patients defines a novel transcriptomic signature of islets from individuals with type 2 diabetes. Diabetologia, *61*(3), 641-657. https://doi.org/10.1007/s00125-017-4500-3
4. Taneera J, Lang S, Sharma A, et al (2012) A systems genetics approach identifies genes and pathways for type 2 diabetes in human islets. Cell Metab, *16*(1), 122-134. https://doi.org/10.1016/j.cmet.2012.06.006
5. Li DS, Yuan YH, Tu HJ, Liang Q Le, Dail LJ (2009) A protocol for islet isolation from mouse pancreas. Nat Protoc, *4*(11), 1649-1652. https://doi.org/10.1038/nprot.2009.150
6. Helman A, Klochendler A, Azazmeh N, et al (2016) p16 Ink4a-induced senescence of pancreatic beta cells enhances insulin secretion. Nat Med, *22*(4), 412-420. https://doi.org/10.1038/nm.4054
7. Kurakula K, Vos M, Rubio IO, et al (2014) The LIM-only protein FHL2 reduces vascular lesion formation involving inhibition of proliferation and migration of smooth muscle cells. PLoS One, *9*(4), e94931. https://doi.org/10.1371/journal.pone.0094931

ELECTRONIC SUPPLEMENTARY MATERIAL (ESM)

ESM TABLES

Table 1 - List of primers used in RT-qPCR

Mouse primers	Forward (5'-3')	Reverse (5'-3')
**18s**	CACTTTTGGGGCCTTCGTG	GCAAAGGCCCAGAGACTCATT
**36b4**	GGACCCGAGAAGACCTCCTT	GCACATCACTCAGAATTTCAATGG
**Atf6**	CCAGATGAAGACTGGGAGTCG	CCCAAGGCATCAAATCCAAATCA
**Bnip3**	AGCTTTGGCGAGAAAAACAG	TGAGAGTAGCTGTGCGCTTC
**Cyclin D2**	AAGCCTGCCAGGAGCAAA	ATCCGGCGTTATGCTGCTCT
**Fhl2**	TCACAGCACGGGATGAGTTTC	GTGCCACCCAGACCACTAATG
**Fos**	AGGGGCAAAGTAGAGCAGCTA	CAATCTCAGTCTGCAACGCA
**Foxa2**	ATTTTAAACCGCCATGCACTCG	GTAGTAGCTGCTCCAGTCGG
**Foxo1**	CTTCAAGGATAAGGGCGACA	GACAGATTGTGGCGAATTGA
**Gcg**	CAGAAGAAGTCGCCATTGCC	GATGAAGTCCCTGGTGGCAA
**Gck**	CACATGTGCTCAGCAGGACT	AGCTTGTACACGGAGCCATC
**Ins2**	GAGCAGGTGACCTTCAGACC	TTCATTGCAGAGGGGTAGGC
**Jun**	TCCCCTATCGACATGGAGTC	TTTTGCGCTTTCAAGGTTTT
**Kir6.2**	CAAGATGCACTTCAGGCAAA	GGTGGGAGGCTTTATGACAA
**Ldha**	CCGTTACCTGATGGGAGAGA	GTAGGCACTGTCCACCACCT
**Mafa**	CAAGGAGGAGGTCATCCGAC	TCTCCAGAATGTGCCGCTG
**Nr4a1 (Nur77)**	ATGCCTCCCCTACCAATCTT	TCTGCCCACTTTCGGATAAC
**Pdx1**	CAGTGGGCAGGAGGTGCTTA	GGGCCGGGAGATGTATTTGTT
**Slc2a2 (Glut2)**	AATGGTCGCCTCATTCTTTG	AGCCAACATTGCTTTGATCC
**Snap25**	ATCAGTGGTGGCTTCATCCGCA	TGGCGGAGGTTTCCGATGATGC
**Stx1a**	GATGAGAAGACAAAGGAGGAACTG	ATGAGCGGTTCAGACCTTCC
**Trpm4**	GCAAGTGCTGAGGACTCTGTTG	CCGTTGATGGTTGCTTGTTGGC
**Vamp2**	CACAATCTGGTTCTTTGAGGAG	AGAGACTTCAGGCAGGAATTAG

Table 2 – List of genes (n=1131) that correlate with FHL2 expression in four human islet microarray datasets (GSE38642, GSE54279, GSE76894, and GSE50397).

Gene Names									
A1CF	APC	BMP5	CDC42EP3	CXCL5	EHD4	FAM46C	GLG1	HOMER2	KCNA3
AADAC	APCDD1L	BMPER	CDCA7L	CXorf57	EIF3B	FAM73A	GLP1R	HS3ST1	KCNA5
AASDH	APH1B	BMPR1B	CDH10	CXXC4	EIF4EBP1	FAM83G	GLRA1	HS6ST2	KCNB2
ABAT	APLP1	BSN	CDK2	CYB5D2	EIF5	FAM84B	GLT8D1	HS6ST3	KCNG3
ABCA2	APOBEC1	BTBD3	CDK8	CYFIP2	EIF6	FAM8A1	GLT8D2	HSPA4L	KCNH2
ABCA3	AQP4	BTRC	CDKL2	CYP2U1	ELAVL4	FAS	GM2A	HTR1F	KCNH6
ABCA5	ARAP2	BYSL	CDON	CYR61	ELK3	FBLN7	GMDS	IAPP	KCNJ11
ABCC5	AREG	C10orf2	CELA3B	CYYR1	ELMO2	FBXL2	GNAI1	ICA1L	KCNJ2
ABCC8	ARHGAP27	C11orf71	CEP97	DACH1	ELOVL4	FBXO17	GNAO1	IDS	KCNJ3
ABCG2	ARHGAP6	C14orf132	CERK	DACH2	ELP4	FBXW4	GNAS	IER2	KCNJ6
ABI2	ARHGEF3	C1orf106	CFLAR	DAZAP1	EMB	FERMT1	GNAZ	IER5	KCNK16
ACACA	ARHGEF9	C21orf59	CHD6	DCHS2	EMG1	FFAR1	GNG2	IFI44	KCNK5
ACACB	ARL1	C2orf73	CHD9	DCX	ENAM	FGF12	GNG5	IGF1R	KCNMA1
ACLY	ARL14	C3orf52	CHGB	DDX18	ENO2	FGF14	GNG7	IGFBP3	KCNMB2
ACOT9	ARL15	C6orf141	CHL1	DDX24	ENPP1	FGF2	GNPNAT1	IL11	KCNN4
ACOXL	ARMC2	CACNA1A	CHM	DENND4C	ENPP2	FGF7	GNS	IL15RA	KCNT2
ACRBP	ARNT2	CACNA1C	CHMP2B	DHRS9	ENPP5	FGF9	GPATCH4	IL18	KCTD12
ACSL5	ARNTL2	CACNA1D	CHN1	DIRAS2	EPB41L3	FGFBP1	GPLD1	IL1R1	KHNYN
ACTL6B	ARPC1A	CACNA2D1	CHRNB2	DKK1	EPB41L5	FHL2	GPN1	IL1R2	KIAA0895
ACVR1C	ARX	CACNA2D2	CHST9	DLG2	EPHA2	FJX1	GPR158	IL1RL1	KIAA1107
ADAMTS9	ASAH1	CACNB2	CLCF1	DLG4	EPHB2	FLII	GPR176	IL1RN	KIAA1324
ADAP1	ASAP2	CACNG4	CLCN4	DMXL2	EPHX2	FLNA	GPR19	IL22RA1	KIAA1324L
ADAP2	ASB4	CADPS	CLDN1	DNAJB11	EPM2AIP1	FLNC	GPRASP1	IL23A	KIAA1958
ADCY1	ASS1	CALB1	CLDN4	DNAJC18	EPOR	FLVCR1	GPRC5A	IL33	KIAA2022
ADPGK	ATF2	CAMK1G	CLIP3	DNAJC27	EPS8	FMN2	GPRIN3	IL4R	KIF3C
ADRBK2	ATL1	CAMK2B	CLPS	DNAJC28	EPS8L3	FOSL2	GRAMD2	IL6	KIF5C
AGR2	ATP10D	CAMK2D	CLSTN3	DNAJC4	ERBB2	FOXP2	GRB7	ILDR2	KL
AHCY	ATP2A3	CAND2	CLTB	DNMBP	ERBB3	FPGS	GRHL2	IMP4	KLF12
AHI1	ATP6AP1	CAPG	CMTM7	DNTTIP2	EREG	FRMD3	GRIA2	IMPDH2	KLF4
AIM1	ATP6AP2	CAPN13	CMTM8	DOCK10	ERLEC1	FRMD8	GRIA3	INA	KLF5
AKT3	ATP6V0A1	CAPN2	CNIH2	DOCK3	ERP29	FRY	GRIA4	INPP5F	KLF6
ALCAM	ATP6V1B2	CARD6	CNKSR2	DOK5	ESRRG	FST	GRIK2	IRAK1BP1	KLHDC1
ALDH3B1	ATP8A1	CASP4	CNNM1	DPH2	ETNK2	FSTL5	GRIK5	IRAK2	KLHL3
ALDH5A1	ATP9A	CASR	CNNM2	DPP6	ETS2	FUCA1	GRPEL1	IRF1	KLHL32
ALKBH2	ATRNL1	CAST	CNTN1	DPYSL2	ETV4	FUT3	GSTA4	ISG20	KLK1
ALS2CL	ATXN2	CAV2	CNTN4	DSCAML1	EVC2	G6PC2	GTF2F2	ISL1	KLKB1
AMOTL2	AVPI1	CBFA2T2	COQ10A	DSG2	EXOC2	GABRB3	GTF3C1	ITGA2	KPNA5
AMZ2	B3GALNT1	CBLC	CORO1C	DUSP19	EXOSC5	GABRG2	GUCY1A3	ITGA3	KRT18
ANGEL1	B3GALT2	CC2D2A	CPE	DUSP26	EZR	GAD2	GUCY1B3	ITGA6	KRT19
ANKH	B3GNT3	CCDC158	CPEB3	DUSP4	F2RL1	GALC	GZF1	ITGB4	KRT222
ANKLE2	B3GNT5	CCDC30	CPM	DUSP5	F3	GALE	HADH	ITGB6	KRT7
ANKS6	BACE1	CCDC50	CPNE3	DYNC1I1	FAM105A	GALNT13	HBEGF	ITM2C	KRT8
ANO5	BAIAP2L1	CCDC68	CREBL2	DYNC2H1	FAM107A	GALNT2	HCFC2	ITPR1	KRT80
ANXA1	BAZ1A	CCDC87	CSRNP3	DZIP3	FAM117A	GALNT5	HEPACAM2	JAKMIP1	KSR2
ANXA10	BBS9	CCL7	CTBS	EDARADD	FAM120C	GARNL3	HEXA	JAKMIP2	KYNU
ANXA2	BCCIP	CCNYL1	CTNNAL1	EDN1	FAM126B	GBP2	HHATL	JAZF1	LAD1
ANXA2P2	BCL10	CCRL2	CTRC	EFCAB7	FAM127A	GCKR	HK1	JOSD1	LAMA3
ANXA3	BCL2L15	CD200	CTSE	EFEMP1	FAM169A	GCNT1	HMGA1	JUP	LAMB3
ANXA5	BEST3	CD58	CXCL16	EFNA1	FAM171B	GDAP1	HMGCLL1	KATNAL1	LAMC2
ANXA6	BHLHB9	CD99L2	CXCL17	EFTUD2	FAM184A	GHSR	HMP19	KBTBD6	LAMP2
AP3B2	BMP2	CDC42EP2	CXCL3	EGFR	FAM19A4	GLCCI1	HNRNPAB	KBTBD7	LAP3

LAS1L	MCOLN3	NEBL	PAQR5	PLCXD3	PUS7	RPH3AL	SGCB	SORL1	TAGLN3
LDHA	MDH1B	NEK6	PARD3	PLD3	QDPR	RPL29	SGSM1	SOX5	TAP1
LDLRAP1	MEF2A	NEO1	PARM1	PLEK2	QPCT	RPS5	SH2D3A	SOX6	TAP2
LDOC1	MEGF9	NET1	PARVB	PLEKHA6	RAB26	RPS6KA4	SH2D4A	SP4	TAPT1
LGALS3	MEIS2	NEUROD1	PAX6	PLEKHB1	RAB27B	RPS6KA6	SH3BGRL	SPATS2L	TBC1D19
LGALS4	MET	NFASC	PBX3	PLXNC1	RAB39B	RPUSD4	SH3BP4	SPHK1	TBC1D30
LHPP	MGAT4C	NFKBIA	PCDH1	PMAIP1	RAB3A	RRAGB	SH3GL2	SPNS2	TBL1X
LIF	MGLL	NIP7	PCDH17	PNMA2	RAB3C	RRAGD	SH3RF2	SPRR3	TCEANC
LIG4	MGP	NIPAL1	PCLO	PNMAL1	RAB9B	RRP15	SHISA2	SPSB1	TCTN1
LINGO2	MIA2	NISCH	PCSK1	PNO1	RABGAP1	RSL1D1	SIDT1	SQRDL	TDRD9
LLPH	MICA	NKX2-2	PCSK2	PNP	RALB	RTN1	SIK2	SRD5A1	TEAD4
LMO4	MICALL1	NKX6-1	PCYOX1	POLD4	RALGPS1	RUNDC3A	SIM1	SRGAP3	TES
LNPEP	MMP7	NLE1	PCYOX1L	POLE4	RAPGEF4	RUNX1T1	SLC16A5	SSBP2	TFF1
LRCH2	MMRN1	NLGN1	PDE3B	POPDC3	RASA3	RXRG	SLC16A7	SSRP1	TFF2
LRFN2	MOCOS	NLK	PDE4DIP	PPARD	RASEF	S100A10	SLC16A9	SST	TFPI
LRRC36	MPHOSPH9	NMNAT2	PDE8B	PPFIA3	RASGEF1A	S100A14	SLC17A5	SSTR1	TFPI2
LRRC42	MPP1	NMNAT3	PDGFC	PPM1E	RASGRF1	S100A16	SLC1A1	SSTR2	TGFA
LRRC49	MPP2	NOL4	PDK3	PPM1G	RASSF8	S100A6	SLC1A5	ST18	TGFBR2
LRRC59	MPZL1	NOP14	PDLIM7	PPM1K	RAVER2	SALL2	SLC22A15	ST3GAL5	TGFBR3
LRRC8D	MPZL2	NOSTRIN	PDX1	PPM1L	RBM3	SAMD3	SLC22A17	ST3GAL6	TGIF1
LRRFIP1	MRAP2	NOVA1	PDZD2	PPP1CA	RBP4	SAMD4A	SLC22A3	ST6GALNAC5	TGIF2
LRRN3	MRPS22	NPC1L1	PEBP1	PPP1R14B	RCAN2	SAP30BP	SLC24A1	ST8SIA3	THSD4
LYAR	MRTO4	NR3C2	PEG10	PPP1R1A	RCAN3	SCAI	SLC25A12	ST8SIA4	TIMM17A
LYSMD2	MSH3	NRCAM	PEPD	PPP1R9A	RCBTB2	SCAMP1	SLC25A15	STEAP4	TINAGL1
MACROD2	MSN	NRSN2	PES1	PPP2R2C	RCC2	SCAMP5	SLC29A4	STK17A	TJP2
MADD	MST1R	NRXN1	PEX1	PPT1	RCOR3	SCAPER	SLC2A12	STK39	TKT
MAFB	MTMR7	NSFL1C	PFKFB2	PRAF2	REEP2	SCD	SLC30A4	STON2	TM4SF1
MAFF	MTSS1	NSUN2	PFKFB3	PRELID2	RELB	SCG2	SLC30A8	STX4	TMC7
MAGED1	MTUS2	NT5DC3	PFN2	PRKACB	RELN	SCG3	SLC35F2	STXBP1	TMCC3
MAGEE1	MUC6	NUCB1	PGAP1	PRKAR2B	RFX3	SCG5	SLC35F3	STXBP4	TMED8
MAGEL2	MUT	NUDT7	PGR	PRKCH	RFX6	SCGN	SLC35F4	STXBP5L	TMEM107
MAGI2	MYC	NXT2	PGRMC2	PRMT3	RGAG4	SCML2	SLC38A4	SULT2B1	TMEM108
MALL	MYH10	OAF	PHLDA1	PROX1	RGL1	SCN2A	SLC46A3	SURF4	TMEM132B
MAMDC2	MYH16	OASL	PHTF2	PRRT3	RGS4	SCN3A	SLC4A8	SUSD1	TMEM17
MAP1LC3A	MYO16	OBFC1	PI3	PRSS8	RGS7	SCN3B	SLC5A6	SUSD4	TMEM171
MAP2K3	MYO1E	OCRL	PIAS1	PRUNE	RGS7BP	SCN8A	SLC6A20	SV2A	TMEM196
MAP6	MYO5A	OGDHL	PIAS2	PRUNE2	RGS9	SCNN1A	SLC6A4	SVIL	TMEM232
MAP7D2	MYO5B	OGN	PIGL	PSD	RHBDD2	SCRN1	SLC7A11	SVOP	TMEM25
MAPK10	MYOF	OSBPL3	PIGP	PSMB7	RHOC	SCRN3	SLC7A8	SYN1	TMEM55A
MAPKAP1	MYT1	OSBPL6	PIK3R3	PSMD14	RHOF	SDC2	SLC8A1	SYNRG	TMEM59
MAPRE3	MYT1L	OSMR	PIM1	PSMD9	RHOT1	SEMA3C	SLC8A2	SYP	TMEM60
MARCH4	NAAA	OXCT1	PIP4K2A	PTCH1	RHPN2	SEMA5A	SMAD3	SYT11	TMEM63C
MARCH5	NACA	OXGR1	PKP3	PTEN	RIMBP2	SEPT3	SMAD9	SYT13	TMOD1
MARCH6	NALCN	P2RY1	PLAC8	PTGS2	RIMS2	SERPINB1	SMAGP	SYT14	TMOD2
MARCH8	NAP1L3	PABPC4	PLAGL1	PTHLH	RIN2	SERPINB7	SMARCA1	SYT17	TMX4
MARCKSL1	NAPB	PABPC5	PLAUR	PTPN4	RIPK4	SERPINI1	SMOX	SYT4	TNFAIP8
MARK1	NBEA	PAK3	PLCB3	PTPRJ	RNF180	SERTAD1	SMPD1	SYT7	TNFRSF10A
MBD2	NCALD	PAK7	PLCB4	PTPRN	RNF39	SESN1	SNAP91	SYT9	TNFRSF10B
MBOAT2	NCAM1	PAM	PLCD3	PTPRN2	RNFT2	SEZ6L	SNRPE	SYTL2	TNFRSF10D
MCF2L	NCEH1	PANX1	PLCE1	PTPRT	ROBO1	SEZ6L2	SNTB1	TACSTD2	TNFSF11
MCF2L2	NDN	PAPPA2	PLCL2	PTX3	ROBO2	SFN	SNX5	TAF4B	TNIP1

TNIP2	TRIM47	TSPYL5	UBAP1	USP51	WDR17	XRN1	ZKSCAN1	ZNF334	ZNF787
TNKS	TRIM59	TTBK2	UBASH3B	UXS1	WDR4	YBX1	ZMAT4	ZNF385D	ZNF827
TOX	TRIP10	TTC21B	UBE2J2	VAMP4	WDR43	YWHAZ	ZMYM3	ZNF451	ZSWIM5
TP53BP1	TRIP6	TTC7B	UBN2	VASH1	WDR46	ZAK	ZMYND12	ZNF462	
TPBG	TRNAU1AP	TTC8	UCHL3	VASP	WDR7	ZBTB4	ZNF12	ZNF483	
TPD52	TRO	TTC9	UCP2	VAT1L	WDR74	ZC3H6	ZNF204P	ZNF540	
TPM4	TRPC1	TUBA1C	UGCG	VAV3	WNK3	ZDHHC15	ZNF223	ZNF543	
TPP1	TRPM3	TUBB6	UNC80	VPS37A	WNK4	ZDHHC7	ZNF233	ZNF546	
TRAF1	TSHZ1	TUSC3	UPK1B	VPS37B	WNT7A	ZFP14	ZNF248	ZNF569	
TRAF4	TSPAN2	TXNRD1	USP11	VWA5A	WSCD2	ZFP30	ZNF25	ZNF571	
TRIM16	TSPAN7	TYRO3	USP27X	VWDE	WWC1	ZFP90	ZNF280B	ZNF660	
TRIM2	TSPYL4	U2AF1	USP43	WDR1	WWTR1	ZHX3	ZNF284	ZNF781	

a

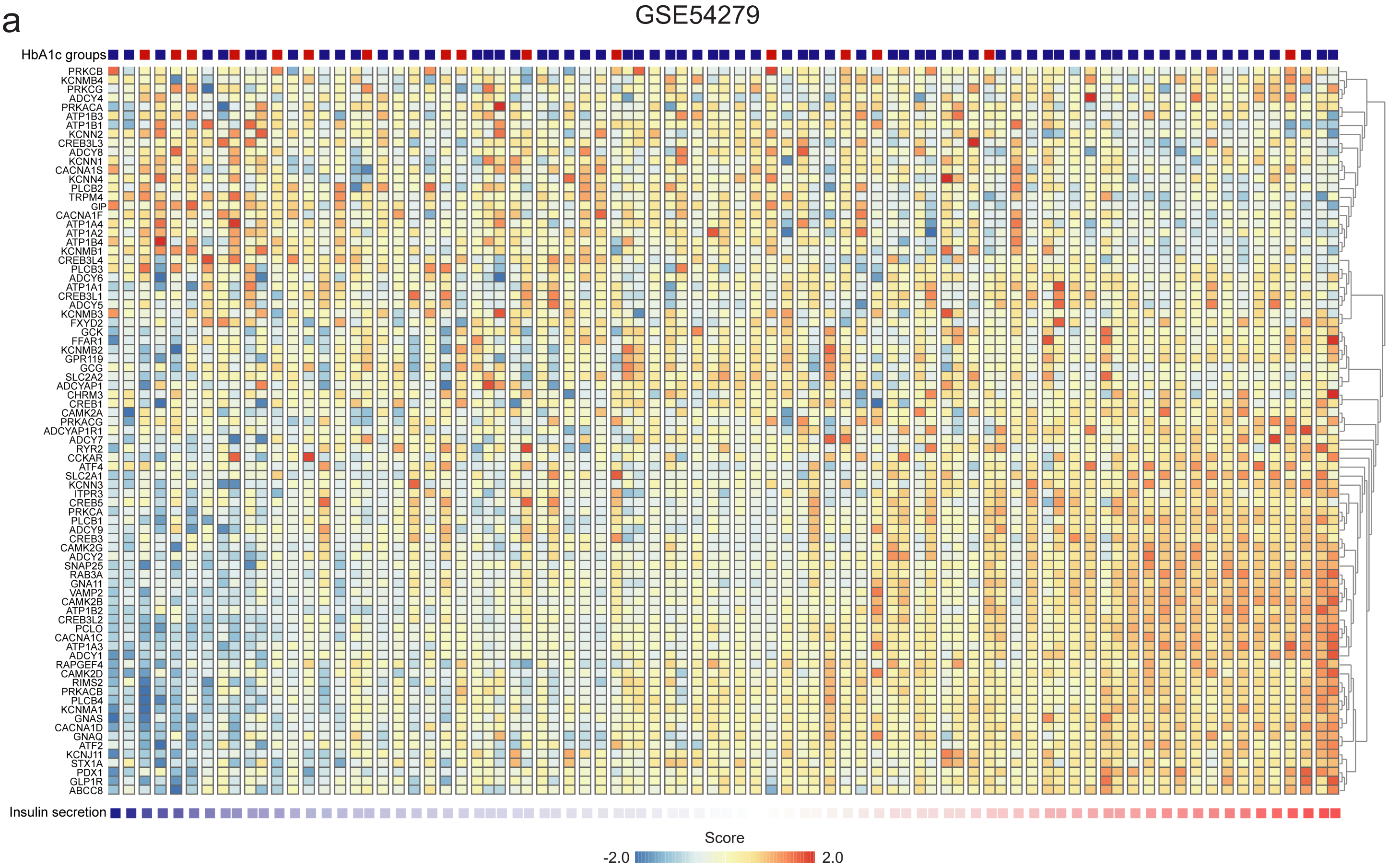

GSE54279
HbA1c groups
PRKCB
KCNMB4
PRKCG
ADCY4
PRKACA
ATP1B3
ATP1B1
KCNN2
CREB3L3
ADCY8
KCNN1
CACNA1S
KCNN4
PLCB2
TRPM4
GIP
CACNA1F
ATP1A4
ATP1A2
ATP1B4
KCNMB1
CREB3L4
PLCB3
ADCY6
ATP1A1
CREB3L1
ADCY5
KCNMB3
FXYD2
GCK
FFAR1
KCNMB2
GPR119
GCG
SLC2A2
ADCYAP1
CHRM3
CREB1
CAMK2A
PRKACG
ADCYAP1R1
ADCY7
RYR2
CCKAR
ATF4
SLC2A1
KCNN3
ITPR3
CREB5
PRKCA
PLCB1
ADCY9
CREB3
CAMK2G
ADCY2
SNAP25
RAB3A
GNA11
VAMP2
CAMK2B
ATP1B2
CREB3L2
PCLO
CACNA1C
ATP1A3
ADCY1
RAPGEF4
CAMK2D
RIMS2
PRKACB
PLCB4
KCNMA1
GNAS
CACNA1D
GNAQ
ATF2
KCNJ11
STX1A
PDX1
GLP1R
ABCC8
Insulin secretion
Score
-2.0
2.0

b

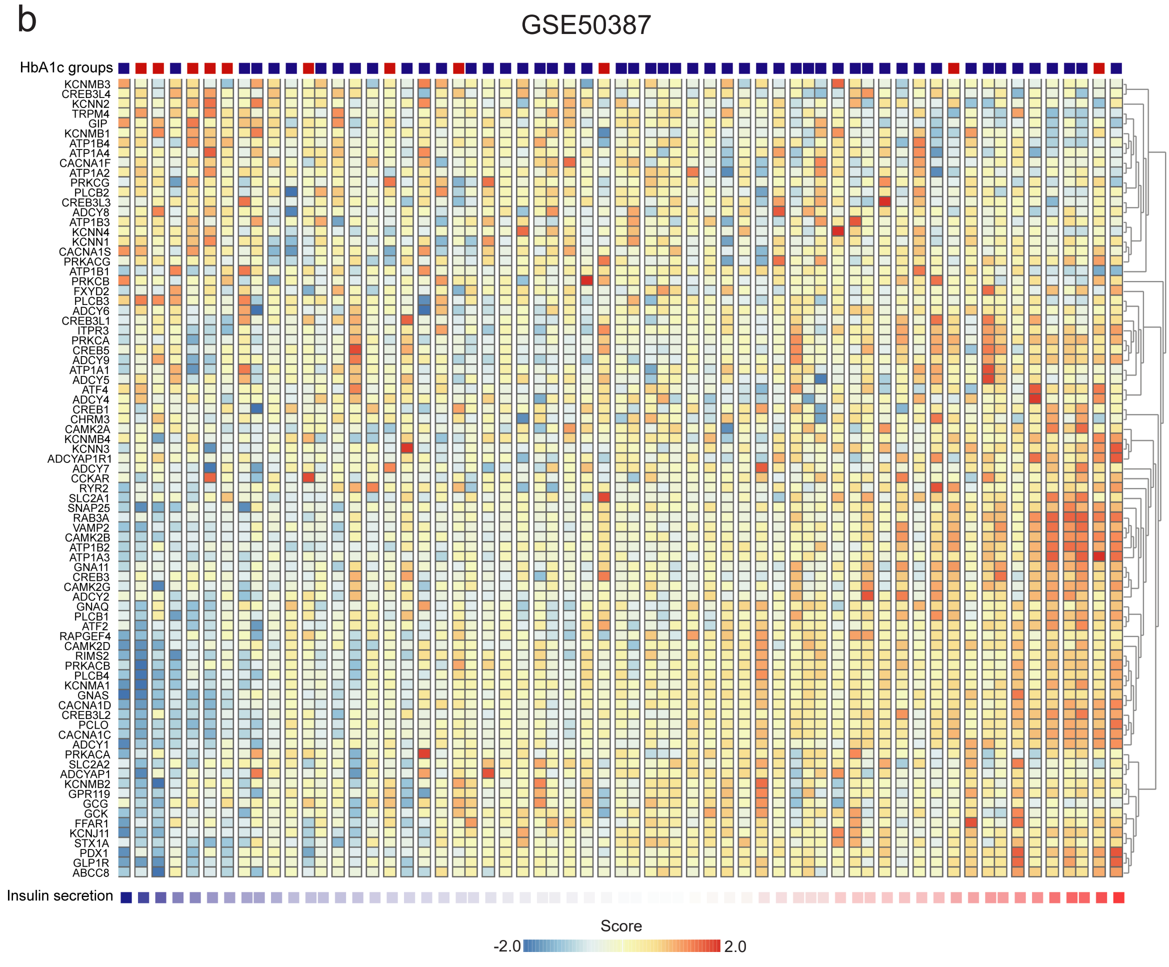

GSE50387
HbA1c groups
KCNMB3
CREB3L4
KCNN2
TRPM4
GIP
KCNMB1
ATP1B4
ATP1A4
CACNA1F
ATP1A2
PRKCG
PLCB2
CREB3L3
ADCY8
ATP1B3
KCNN4
KCNN1
CACNA1S
PRKACG
ATP1B1
PRKCB
FXYD2
PLCB3
ADCY6
CREB3L1
ITPR3
PRKCA
CREB5
ADCY9
ATP1A1
ADCY5
ATF4
ADCY4
CREB1
CHRM3
CAMK2A
KCNMB4
KCNN3
ADCYAP1R1
ADCY7
CCKAR
RYR2
SLC2A1
SNAP25
RAB3A
VAMP2
CAMK2B
ATP1B2
ATP1A3
GNA11
CREB3
CAMK2G
ADCY2
GNAQ
PLCB1
ATF2
RAPGEF4
CAMK2D
RIMS2
PRKACB
PLCB4
KCNMA1
GNAS
CACNA1D
CREB3L2
PCLO
CACNA1C
ADCY1
PRKACA
SLC2A2
ADCYAP1
KCNMB2
GPR119
GCG
GCK
FFAR1
KCNJ11
STX1A
PDX1
GLP1R
ABCC8
Insulin secretion
Score
-2.0
2.0

c

GSE76894

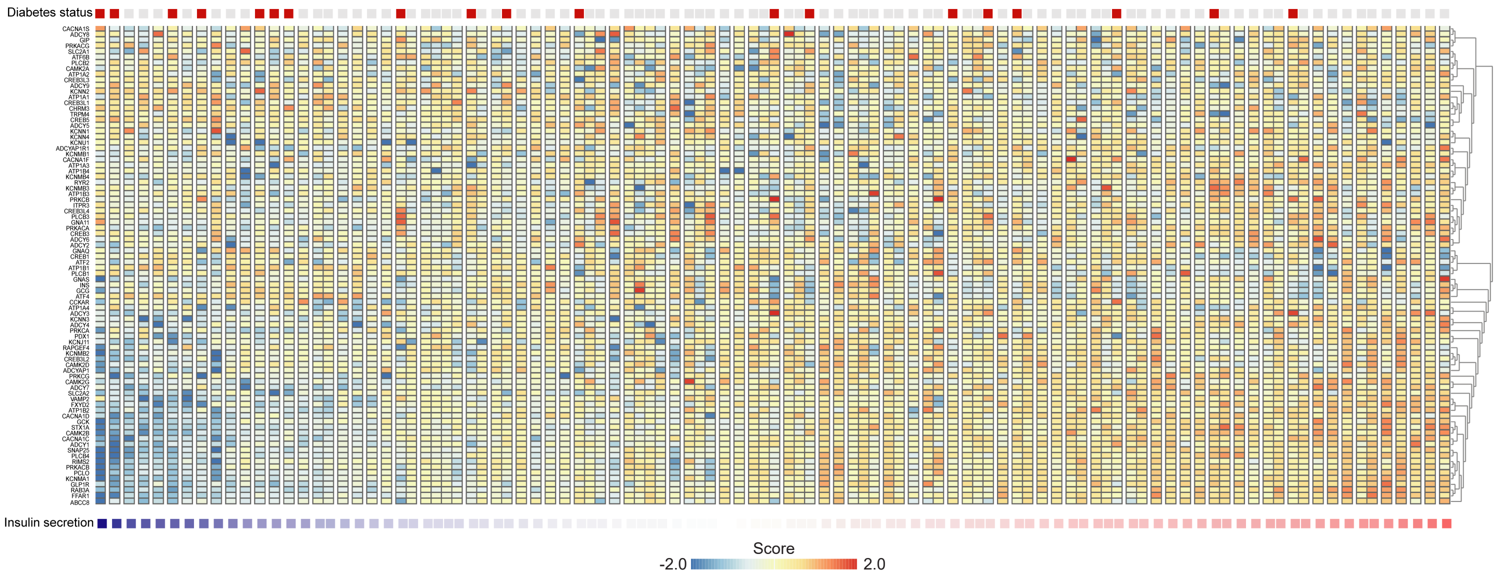

d

GSE38642

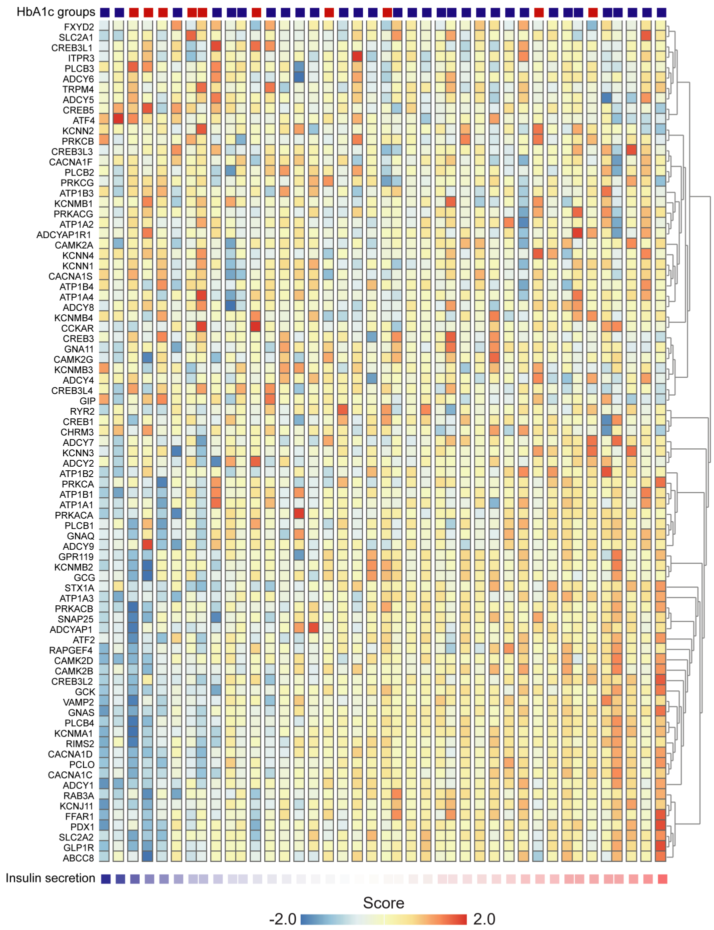

e

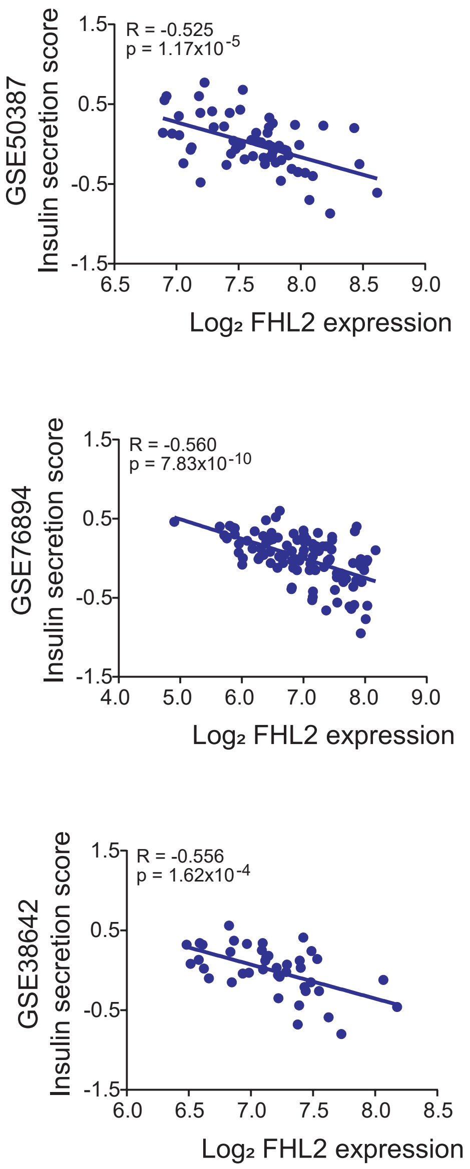

Figure 1: Heatmap of genes expressed in the insulin secretion pathway from human pancreatic islets datasets.
a: Dataset GSE54279 organized by insulin secretion score. Samples on the top divided in low (blue) and high (red) HbA1c levels. **b:** Dataset GSE50387 organized by insulin secretion score. Samples on the top divided in low (blue) and high (red) HbA1c levels. **c:** Dataset GSE76894 organized by insulin secretion score. Samples on the top divided in non-disease individuals (grey) and individuals with type 2 diabetes (red). **d:** Dataset GSE38642 organized by insulin secretion score. Samples on the top divided in low (blue) and high (red) HbA1c levels. **e:** Correlation of insulin secretion pathway signature score and FHL2 expression in datasets GSE50387, GSE76894 and GSE38642.

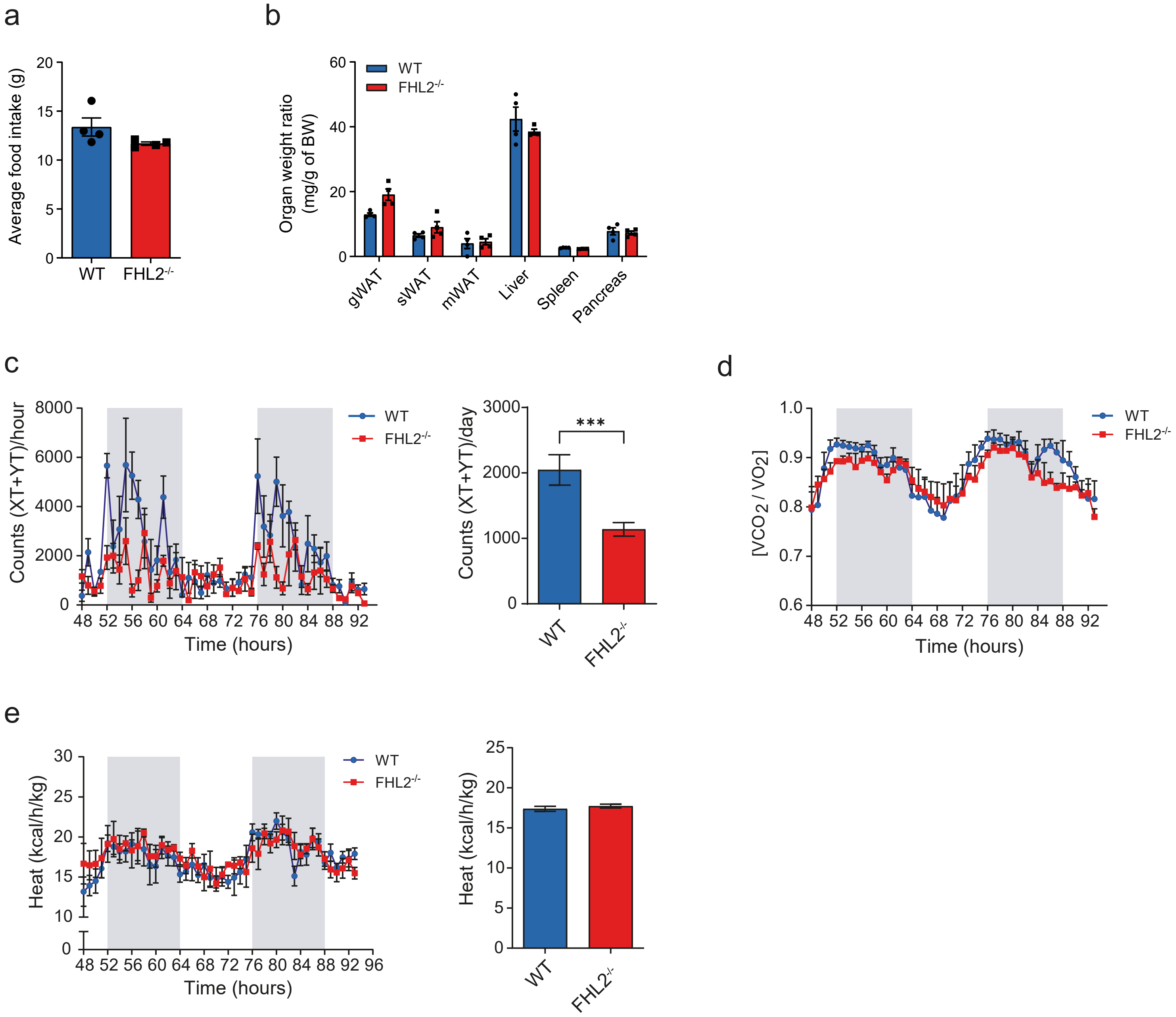

Figure 2: Indirect calorimetry showed no differences in energy metabolism between WT and FHL2$^{-/-}$ mice.
a: Average food intake of WT and FHL2$^{-/-}$ mice during the five days of metabolic cage experiment (n=4; for all the graphs). **b:** Organ weight (as mg per g of bodyweight) WT and FHL2$^{-/-}$ mice. **c:** Locomotor activity per hour (measured as XT +YT movement in cage) of WT and FHL2$^{-/-}$ mice during metabolic cage experiment and average per day. **d:** Respiratory exchange ratio (RER) of WT and FHL2$^{-/-}$ mice. **e:** Energy expenditure or heat (kcal/h/kg) of WT and FHL2$^{-/-}$ mice and average.
Data are indicated as mean ± SEM (*$p<0.05$).

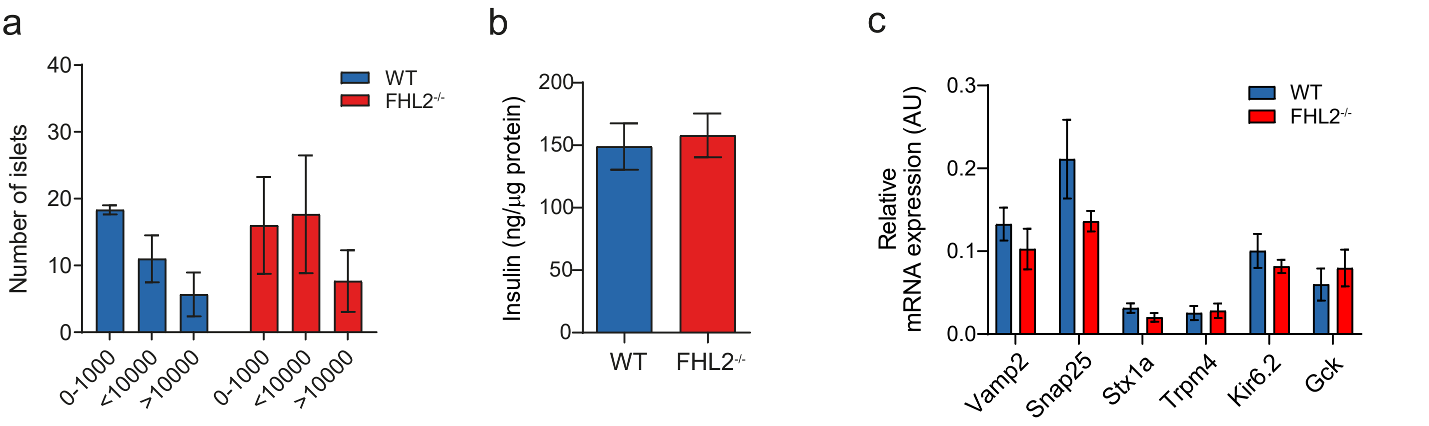

Figure 3: Islet size and insulin content is similar between WT and FHL2$^{-/-}$ mice.
a: Islet size distribution from immunofluorescence staining of WT and FHL2$^{-/-}$ pancreas (n=3). **b:** Total insulin content of isolated pancreatic islets from WT (n=27) and FHL2$^{-/-}$ (n=29) mice. **c:** Relative mRNA expression of other genes in isolated WT and FHL2$^{-/-}$ pancreatic islets. Data are indicated as mean ± SEM (*p<0,05).

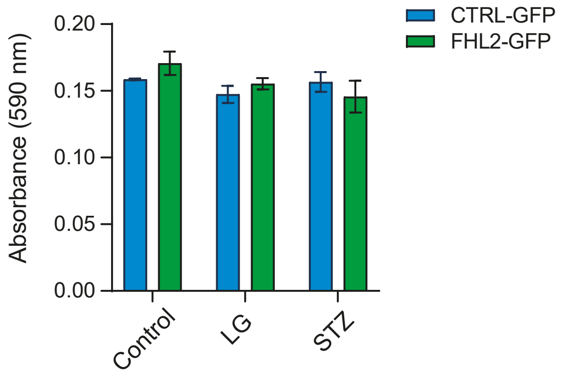

Figure 4: MIN6 cells overexpressing FHL2-GFP show similar cell viability as control cells.
MTT cell viability assay on MIN6 cells overexpressing CTRL-GFP and FHL2-GFP (n=3). Data are indicated as mean ± SEM (*p<0,05).
